# Argonaute 5 family proteins play crucial roles in the defence against *Cymbidium* *mosaic* *virus* and *Odontoglossum* *ringspot* *virus* in *Phalaenopsis* *aphrodite* subsp. *formosan* *a*

**DOI:** 10.1111/mpp.13049

**Published:** 2021-03-21

**Authors:** Song‐Yi Kuo, Chung‐Chi Hu, Ying‐Wen Huang, Chin‐Wei Lee, Meng‐Jhe Luo, Chin‐Wei Tu, Shu‐Chuan Lee, Na‐Sheng Lin, Yau‐Heiu Hsu

**Affiliations:** ^1^ Institute of Plant and Microbial Biology Academia Sinica Taipei Taiwan; ^2^ Graduate Institute of Biotechnology National Chung Hsing University Taichung Taiwan; ^3^ Advanced Plant Biotechnology Center National Chung Hsing University Taichung Taiwan; ^4^ Microbial Genomic National Chung Hsing University and Academia Sinica Taichung Taiwan

**Keywords:** antiviral RNA silencing, Argonaute protein, *Cymbidium mosaic virus*, *Odontoglossum ringspot virus*, *Phalaenopsis aphrodite* subsp. *formosana*, virus‐induced gene silencing (VIGS)

## Abstract

The orchid industry faces severe threats from diseases caused by viruses. Argonaute proteins (AGOs) have been shown to be the major components in the antiviral defence systems through RNA silencing in many model plants. However, the roles of AGOs in orchids against viral infections have not been analysed comprehensively. In this study, *Phalaenopsis aphrodite* subsp. *formosana* was chosen as the representative to analyse the AGOs (PaAGOs) involved in the defence against two major viruses of orchids, *Cymbidium mosaic virus* (CymMV) and *Odontoglossum ringspot virus* (ORSV). A total of 11 PaAGOs were identified from the expression profile analyses of these PaAGOs in *P. aphrodite* subsp. *formosana* singly or doubly infected with CymMV and/or ORSV. PaAGO5b was found to be the only one highly induced. Results from overexpression of individual PaAGO5 family genes revealed that PaAGO5a and PaAGO5b play central roles in the antiviral defence mechanisms of *P. aphrodite* subsp. *formosana*. Furthermore, a virus‐induced gene silencing vector based on *Foxtail mosaic virus* was developed to corroborate the function of PaAGO5s. The results confirmed their importance in the defences against CymMV and ORSV. Our findings may provide useful information for the breeding of traits for resistance or tolerance to CymMV or ORSV infections in *Phalaenopsis* orchids.

## Graphical abstract

*Phalaenopsis aphrodite* subsp. *formosana* harbours 11 argonaute genes (*PaAGOs*), among which *PaAGO5b* plays a crucial role in the antiviral defence mechanisms against *Cymbidium mosaic virus* and *Odontoglossum ringspot virus*.

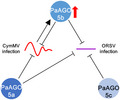

## 1 INTRODUCTION

Orchidaceae is considered the largest vascular plant family, representing more than 35,000 species in about 880 genera (Wang et al., 2016). Orchids are among the most valuable ornamental crops in horticulture and the floral industry. However, cultivation and marketability of orchids have been greatly hampered by various pathogens, especially viruses that are not effectively controlled by pesticide applications. Orchids have been reported to be infected by more than 50 species of viruses, among which Cymbidium mosaic virus (CymMV) and Odontoglossum ringspot virus (ORSV) are two of the most prevalent viral pathogens that have posed serious threats to the orchid industry.

CymMV and ORSV both contain a monopartite, positive‐sense RNA genome. *Cymbidium mosaic virus* belongs to the genus *Potexvirus* with a genomic RNA that is around 6,227 nucleotides (nt) in length and contains five open reading frames that encode an RNA‐dependent RNA polymerase (RdRp), three triple‐gene‐block proteins (TGBps), and a capsid protein (CP) (Wong et al., 1997). *Odontoglossum ringspot virus* is a member of the genus *Tobamovirus* and has a genomic RNA of about 6,600 nt in length that encodes the 126K/183K proteins for viral replication, a movement protein, and a CP (Ryu & Park, 1995). The symptom caused by CymMV and/or ORSV usually varies in different species of orchids and is often confused with those induced by other factors, such as abiotic stress, malnutrition, and phytotoxicity (Moraes et al., 2017). The orchid species of *Cymbidium*, *Cattleya*, *Epidendrum*, and *Phalaenopsis* are considered to be more susceptible to CymMV infection. In contrast, ORSV is reported to be more infectious on *Odontoglossum grande* and *Cymbidium alexanderi* and the symptoms of ORSV‐infected *Phalaenopsis amabilis* may be less evident (Ajjikuttira & Wong, 2009; Lai et al., 2013). Coinfection of CymMV and ORSV on the same plant, commonly observed in the plantations, usually leads to synergistic effects and, as a result, higher viral accumulation as well as more severe symptoms (Chen et al., 2019; Hu et al., 1998; Pearson & Cole, 1991). The differences in symptoms caused by individual or a combination of viruses suggest that different host factors might be involved in symptom development and/or host defence responses. However, the factors and mechanisms involved await further exploration.

Among the host factors associated with viral infection processes and defence responses, argonaute proteins (AGOs) have attracted much attention in recent studies. AGOs are the key components of RNA‐induced silencing complexes (RISCs) and play a major role in RNA silencing (Carbonell & Carrington, 2015; Fang & Qi, 2016; Zhang et al., 2015). Plants encode numerous AGOs that are involved in the developmental programming, responses to biotic and abiotic stresses, DNA repair, antiviral defences, and other regulatory mechanisms. Based on phylogenetic relationships, the AGO family could be subdivided into four clades: AGO1/10, AGO2/3/7, AGO4/6/8/9, and AGO5/12/13/14 (Rodríguez‐Leal et al., 2016). The members of the AGO4/6/8/9 clade are mostly involved in RNA‐directed DNA methylation (RdDM) activities that function in the nucleus to mediate epigenetic modification and regulate gene expression (Matzke et al., 2015). AGO2/3/7, AGO1/10, and AGO5 clades are generally associated with the posttranscriptional gene silencing pathway or translational repression of target RNAs (Carbonell et al., 2012; Fátyol et al., 2015; Iwakawa & Tomari, 2013; Shao et al., 2014).

Many AGOs have been reported to mediate defence against viruses. For example, in *Arabidopsis thaliana*, AGO1 participates in the defence mechanism for restricting turnip crinkle virus (TCV) and cucumber mosaic virus (CMV) infections (Qu et al., 2008; Wang et al., 2011). In *Nicotiana benthamiana*, NbAGO1 was recently found to inhibit bamboo mosaic virus (BaMV) accumulation, and NbAOG10 may compete with NbAGO1 for BaMV‐derived small interfering RNAs (vsiRNAs) to protect BaMV from NbAGO1‐mediated antiviral RNA cleavage (Huang et al., 2019). AGO2 is involved in the defence against potato virus X (PVX), TCV, CMV, and turnip mosaic virus (TuMV) in various plants (Harvey et al., 2011). The abscisic acid‐mediated up‐regulation of AGO2 and AGO3 induces resistance to BaMV (Alazem et al., 2017). The deficiency of AtAGO4 predisposes *A. thaliana* plants to tobacco rattle virus (TRV) infection (Ma et al., 2015). AtAGO7 and AtAGO10 provide resistance against TCV and TuMV, respectively (Garcia‐Ruiz et al., 2015; Qu et al., 2008). AGO18 is a grass‐specific AGO subfamily and is close to the AGO1/5/10 clade. OsAGO18 from *Oryza sativa* was reported to be induced in rice infected by rice dwarf virus (RDV) and rice stripe virus (RSV), and is involved in the maintenance of OsAGO1 expression through the sequestration of miR168, which in turn improves the OsAGO1‐mediated antiviral defence against RDV and RSV (Wu et al., 2015). Despite the wealth of studies on the importance of AGOs in antiviral mechanisms in the model plants mentioned above, the identities and functions of AGOs against viral infections in orchids have not been systematically analysed previously.

In this study, *Phalaenopsis aphrodite* subsp. *formosana,* the moth orchid native to Taiwan, was chosen as the representative orchid, based on economic importance and the availability of the genomic information (Chao et al., 2017), for the comprehensive analysis of the functions of AGOs against viral infections. Our previous study revealed that the accumulation levels of four AGOs (PaAGO1, 4, 5, and 10) in leaves varied significantly depending on the invading viruses (Pai et al., 2020). Here, we identified and analysed the expression profiles of all candidate PaAGOs in response to different viral infections. Subsequently, the PaAGO5 family proteins were targeted for further analysis on their roles in antiviral mechanisms by gene knockdown or overexpression because PaAGO5s were the only ones found to be responsive to viral infections in this study. It was revealed that both PaAGO5a and PaAGO5b could enhance resistance against CymMV and ORSV. To our knowledge, this is the first comprehensive study of the AGOs in response to virus infections in *P. aphrodite* subsp. *formosana*, demonstrating the unique involvement of PaAGO5 family proteins against prevalent orchid viruses.

## 2 RESULTS

### 2.1 Identification of the AGOs in *P. aphrodite* subsp. *formosana*

**FIGURE 1:**
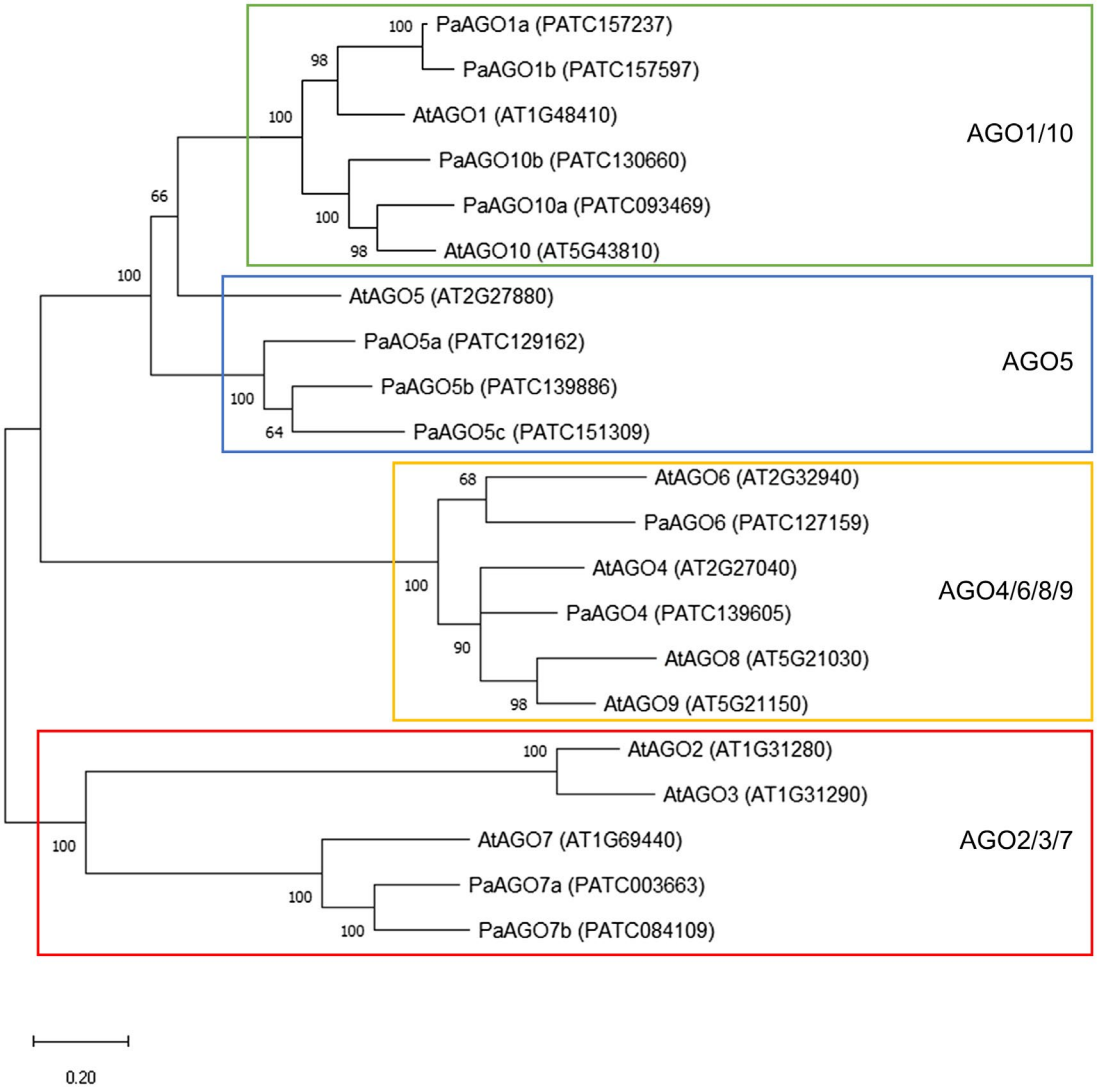
Phylogenetic relationship of the Argonaute (AGO) family proteins of *Phalaenopsis aphrodite* subsp. *formosana*. The amino acid sequences of AGO proteins identified in *P. aphrodite* subsp. *formosana,* with those from *Arabidopsis thaliana* as references, were aligned using the ClustalW software (Thompson et al., 1994) and subjected to phylogenetic analysis using the maximum‐likelihood method in MEGA software (Kumar et al., 2018) with 1,000 bootstrapping replicates using default parameters. The bootstrap values are shown next to the branches. Major clades are boxed in different colours according to the classification reported by Rodriguez‐Leal et al. (2016). Scale bar, 0.2 substitutions per site

Although the transcriptomic information of *P. aphrodite* subsp. *formosana* is available (Chao et al., 2017), the genes encoding AGOs (PaAGOs) have not been thoroughly identified previously. To provide a comprehensive understanding of the AGOs involved in the defence responses against viruses, we performed exhaustive searches in different genomic databases using the sequences of known AGO genes from *N. benthamiana* and *Arabidopsis thaliana* as queries with the BLAST search (Altschul et al., 1990). The databases searched included GenBank (www.ncbi.nlm.nih.gov/genbank, Sayers et al., 2020) of the National Center for Biotechnology Information (NCBI) and Orchidstra 2.0 (Chao et al., 2017). In addition, keyword searches of annotated AGOs were also performed in these databases. A total of 11 PaAGOs were identified from the Orchidstra 2.0 database for orchid transcriptomes. To illustrate the evolutionary relationship with the AGOs of known model plants, we conducted a multiple sequence alignment using ClustalW (Thompson et al., 1994) followed by phylogenetic analysis of the 11 PaAGOs and representatives from *A. thaliana* (AtAGOs) (Berardini et al., 2015), using the general time reversible (GTR; Tavaré, 1986) substitution model and the maximum‐likelihood method for phylogenetic tree reconstruction in MEGA X (Adachi et al., 2000; Kumar et al., 2018). In accordance with previous reports (Huang et al., 2019; Rodríguez‐Leal et al., 2016), the 11 PaAGOs were grouped into AGO1, AGO4, AGO5, AGO6, AGO7, and AGO10 with the previous classification of AtAGOs subfamilies (Figure 1). The designations of the transcripts were as follows: PATC157237 (PaAGO1a), PATC157597 (PaAGO1b), PATC129162 (PaAGO5a), PATC139886 (PaAGO5b), PATC151309 (PaAGO5c), PATC139605 (PaAGO4), PATC127159 (PaAGO6), PATC003663 (PaAGO7a), PATC084109 (PaAGO7b), PATC130660 (PaAGO10a), and PATC093469 (PaAGO10b). From the transcript expression data in the Orchidstra 2.0 database, PaAGO1a showed the highest expression level among all PaAGOs in leaves (Figure S1a). PaAGO1b and PaAGO4a exhibited a similar expression level, followed by PaAGO5b, which is the most abundant PaAGO5. The expression level of the rest of the PaAGOs in descending order was PaAGO10b, PaAGO6, PaAGO10a, PaAGO7a, and PaAGO7b. The tissue‐specific expression profile indicated that most of the PaAGOs were primarily distributed in flower, root, stalk, or seed (Figure S1b), with lower expression levels in leaf and polonium.

### 2.2 PaAGOs expression profiles following CymMV and/or ORSV infection

**FIGURE 2:**
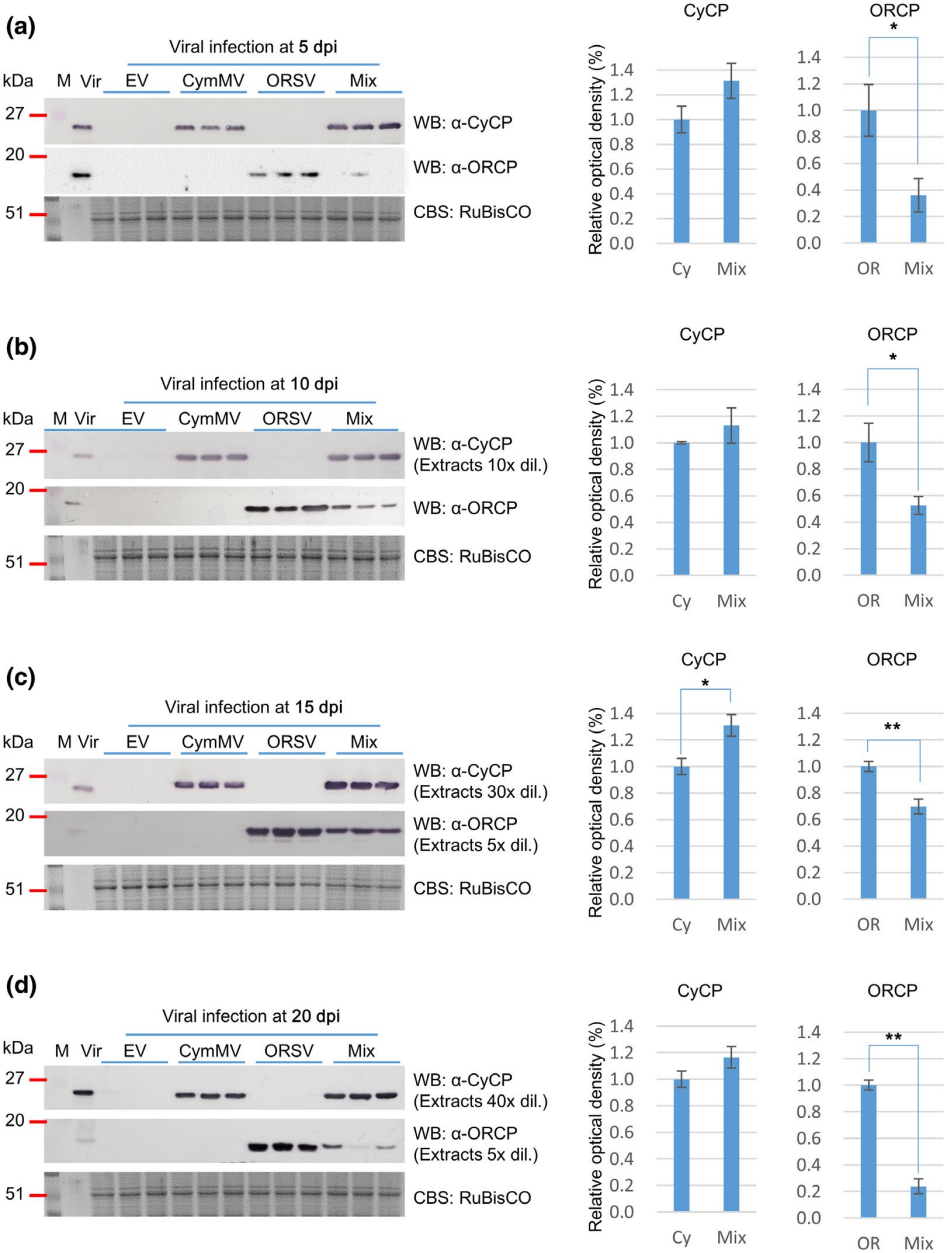
Accumulation of viral coat proteins (CP) at different intervals following infections by CymMV and/or ORSV in *Phalaenopsis aphrodite* subsp. *formosana* leaves. *P. aphrodite* subsp. *formosana* leaves were infiltrated with *Agrobacterium tumefaciens* EHA105 harbouring infectious clones of CymMV and ORSV, pKCy1 and pKORy‐15–2, respectively, using the AGROBEST method (Wu et al., 2014), either alone or mixed, at an OD_600_ of 0.5. The leaves were collected at 5 (a), 10 (b), 15 (c), and 20 (d) days postinoculation (dpi) for analysis. The protein extracts from leaves at different dilutions were electrophoresed through a 12.5% acrylamide gel containing 1% sodium dodecyl sulphate (SDS‐PAGE), followed by western blot (WB) analysis using specific antibodies against CymMV or ORSV as indicated. The samples were diluted differently, as indicated, to accommodate the differences of each antibody. The intensity of each band was quantified and plotted. Coomassie blue‐stained RuBisCO protein (CBS‐RuBisCO) was used as the loading control. Samples from *P. aphrodite* subsp. *formosana* leaves agroinfiltrated with empty vector pKn only (EV), pKCy1 alone (CymMV), pKORy‐15–2 alone (ORSV), or both infectious clones (Mixed) were as indicated. Vir, the mixture of virions containing 50 ng CymMV and 5 ng ORSV

**FIGURE 3:**
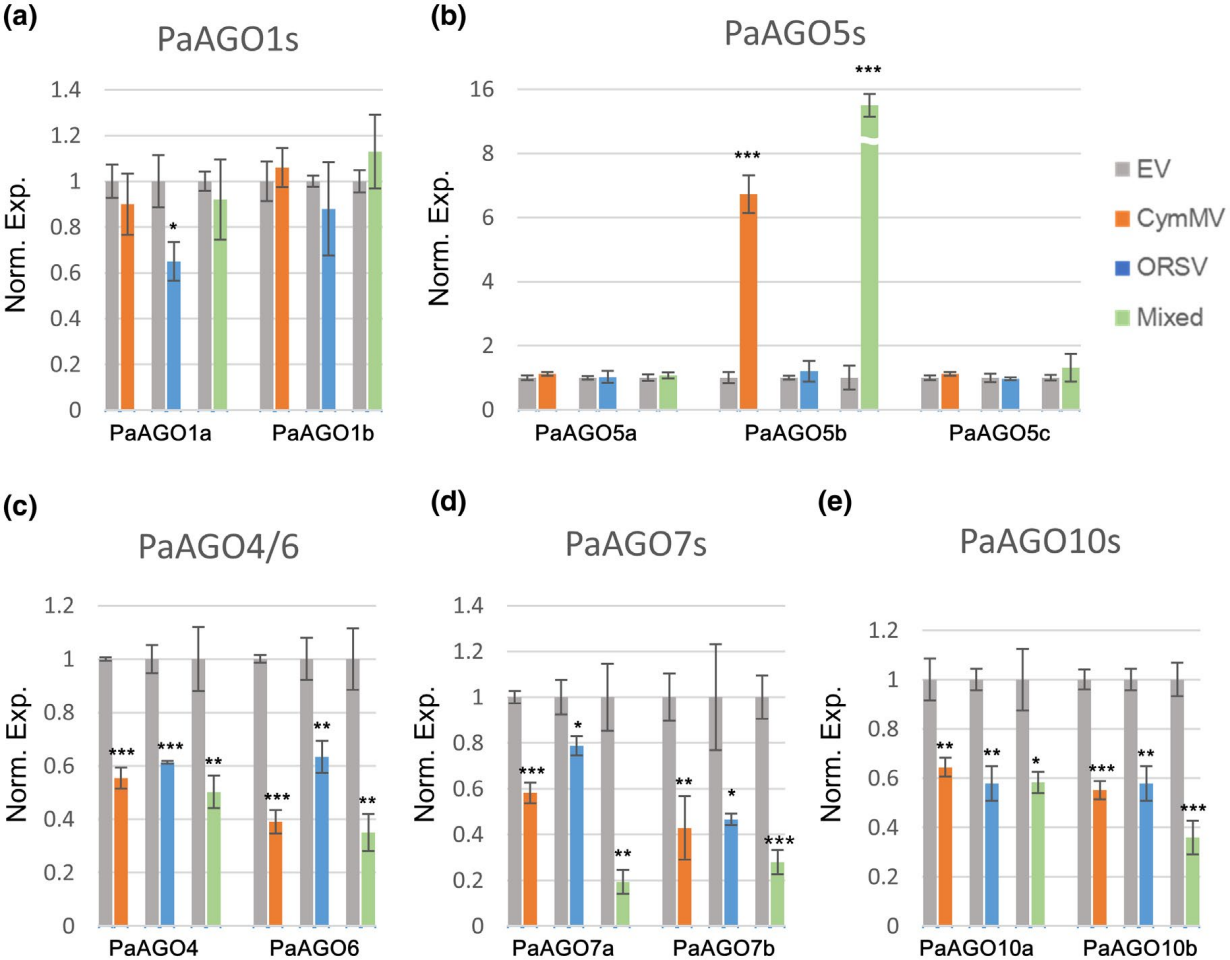
The expression profile of *PaAGO*s in virus‐infected leaves. The leaves of *Phalaenopsis aphrodite* subsp. *formosana* were inoculated with infectious clones of CymMV, ORSV, or both via agroinfiltration (indicated as CymMV, ORSV, and Mixed, respectively). The leaves were collected at 5 days postinoculation for total RNA extraction. The transcript accumulation levels were examined by quantitative reverse transcription PCR. The expression levels of each PaAGO transcript, presented as normalized fold changes relative to that from mock‐inoculated leaves (EV), are shown. (a) PaAGO1a and PaAGO1b, (b) PaAGO4 and PaAGO6, (c) PaAGO7a and PaAGO7b, (d) PaAGO5a, PaAGO5b, and PaAGO5c, and (e) PaAGO10a and PaAGO10b. Values are means ± *SD* of three biological replicates. Norm. Exp, normalized expression level; *, ** and ***, significant difference at *p* < .05, *p* < .01, and *p* < .001 determined by Student's *t* test, respectively

To unveil the roles of the PaAGOs in the antiviral defence responses, the expression profiles of PaAGOs during viral infections were examined. *P. aphrodite* subsp. *formosana* leaves at the four‐ to five‐leaf stages were inoculated with CymMV and/or ORSV infectious clones through agroinfiltration. The plants were kept at 28 °C with a 15‐hr light period and leaf samples were collected at 5, 10, 15, and 20 days postinoculation (dpi). The accumulation levels of *PaAGO* transcripts and viruses were analysed by quantitative reverse transcription‐polymerase chain reaction (RT‐qPCR) and western blot. The result showed an asymmetric synergism between CymMV and ORSV, similar to that reported previously (Pai et al., 2020). CymMV infection suppressed ORSV accumulation during the entire infection period. In contrast, CymMV accumulation was slightly higher in mix‐infected leaves (Mix) than that in CymMV‐infected leaves at 15 dpi (Figure 2). The PaAGO5b transcript accumulation level exhibited the most prominent increases in CymMV‐ and mix‐infected leaves, by 6.7‐ and 14.5‐fold, respectively (Figure 3b). The increasing level of PaAGO5b was directly proportional to the level of CymMV accumulation, but not affected by ORSV infection. The ORSV infection resulted in negative regulation of the accumulation of PaAGO1a. However, the amounts of PaAGO1b, PaAGO5a, and PaAGO5c transcripts showed no significant differences in the virus‐infected leaves (Figure 3a,b), while the accumulation of the other members of the AGO1/10 clade, PaAGO10a and 10b, was reduced instead (Figure 3e). The amounts of PaAGO4, PaAGO6, PaAGO7a, and PaAGO7b transcripts decreased in all virus‐infected leaves as well (Figure 3c,d). The accumulation of PaAGO7a and PaAGO7b was particularly reduced in mixed infection. In general, the levels of most of PaAGOs significantly declined during viral infections, with a few maintaining the basal transcription levels (PaAGO1b, PaAGO5a, and PaAGO5c). The expression of PaAGO5 has been reported to be induced by the infection of CymMV or mixed infection of CymMV and ORSV (Pai et al., 2020); however, the levels of PaAGO5a, 5b, and 5c were not distinguished in the previous study. The present results further revealed that PaAGO5b may be the actual PaAGO5 that responded to CymMV infection.

### 2.3 Overexpression of PaAGO5s confers resistance to CymMV and ORSV infections in *P. aphrodite* subsp. *formosana*

To further analyse the functions of PaAGO5s in antiviral defence, the coding sequences of all three PaAGO5s were individually cloned. Specific primers for cloning PaAGO5s were designed based on the sequences in the Orchidstra 2.0 database. PaAGO5 coding sequences were amplified using RT‐PCR with respective primer pairs (Table 1) and the 5′ termini of the mRNAs were further examined by 5′ rapid amplification of cDNA ends (5′ RACE). The cDNAs of PaAGO5a (1,965 bp) and PaAGO5b (2,901 bp) share 100% and 99.9% identities with the sequences in the Orchidstra 2.0 database, respectively. The corresponding sequence for PaAGO5c in the database is only 975 nt long; however, our 5′ RACE analysis extended the 5′‐terminal sequence and revealed that PaAGO5c mRNA is 2,040 nt in length. Protein sequence alignment showed that all PaAGO5s comprise the typical PAZ, MID, and PIWI domains with comparable sizes. The size of PaAGO5b is much larger than those of the other PaAGO5s, with an extended N‐terminal domain (Figure S2a). The characteristic DDH catalytic triad, key metal‐coordinating residues involved in RNase H activity (Jullien et al., 2020), and RNA interacting region were conserved in all three PaAGO5s and shared a high sequence similarity (Figure S2b). The above finding suggested that all PaAGO5s may be involved in the RNA silencing machineries.

**TABLE 1:** List of the primers used for quantitative reverse transcription PCR (RT‐qPCR) and the cloning of DNA fragments

For real‐time RT‐qPCR			
Transcript	Sequence (5′—3′)		Product length (bp)
PaAGO1a	AGO1a‐F	GTGGACCTGTTCCTGGTGGT	365
	AGO1 u‐R	CTGGATTCCGGCATGACTGCAC	
PaAGO1b	AGO1b‐F	GGTGGCATGATCGGGGAGCTTC	351
	AGO1 u‐R	CTGGATTCCGGCATGACTGCAC	
PaAGO4	AGO4‐F	CATTGCCTCTTTAGTTTCCC	228
	AGO4‐R	GCGAACCAGTTTCTATCATCAC	
PaAGO5a	AGO5 u‐F	GCTGGTATTCAGGGCACAAG	212
	AGO5a‐R	CCATATTCAGACCCTGCTTCC	
PaAGO5b	AGO5 u‐F	GCTGGTATTCAGGGCACAAG	300
	AGO5b‐R	CATCCCATTCGTAATGTTCTCC	
PaAGO5c	AGO5 u‐F	GCTGGTATTCAGGGCACAAG	325
	AGO5c‐R	CGTTTGCGACGTTTTCTGG	
PaAGO6	AGO6‐F	GCAGATTGGTCAACAAAC	215
	AGO6‐R	CAAGCTCCATATCTTGTCTGC	
PaAGO7a	AGO7a‐F	GCATTTGGCTGCGTATAGAG	261
	AGO7a‐R	CAATTACATTCAATCCCCACC	
PaAGO7b	AGO7b‐F	GGGCTTTGATAAGCTTTGG	163
	AGO7b‐R	GCATGTGCGTTGCTGAGC	
PaAGO10a	AGO10a‐F	CTATGCACACTTAGCAGCC	259
	AGO10a‐R	CAATGCCAAAAGTCCATTC	
PaAGO10b	AGO10b‐F	GCAGAAGAGAGGATGAGAAGAGG	221
	AGO10b‐R	GATGTTTACAGCATAGTCCTC	

For PaAGO5s coding sequence (cds) cloning			
Gene	Sequence^a^, ^b^		Product length (bp)
PaAGO5a	KpnI/PaAGO5a CDSF	GCGGTACC *ATGGATTACAAGGACGACGATGAC*AAGGGCTTATGCTTGAAC	2005
	SacI/PaAGO5a CDSR	GCGAGCTCTTAACAGTAAAACATC	
PaAGO5b	KpnI/PaAGO5b CDSF	GCGGTACC *ATGGACTACAAGGACGATGACGAT*AAGTCTCGTAGCGGCGGTTG	2,941
	SacI/PaAGO5b CDSR	GCGAGCTCCTAACAGTAAAACATCC	
PaAGO5c	KpnI/PaAGO5c CDSF	GCGGTACC *ATGGACTACAAGGACGATGACGAT*AAGTCTGGAGGTTTAGAATGTTG	2080
	SacI/PaAGO5c CDSR	GCGAGCTCCTAACAGTAAAACATCACG	

For PaAGO5u inverted repeat (IR) cloning			
Template	Sequence		Product length (bp)
PaAGO5b cds	PaAGO5 u IR F‐ HpaI	CGGTTAACGAGCGGGAACATATTGCCAG	269
	PaAGO5 u IR R‐ SpeI	CGACTAGTGCTGCAAGATGCGCATAAT	

^a^ Restriction enzyme sites located in primer sequences are underlined.

^b^ FLAG‐tags located in primer sequences are shown in italics.

**FIGURE 4:**
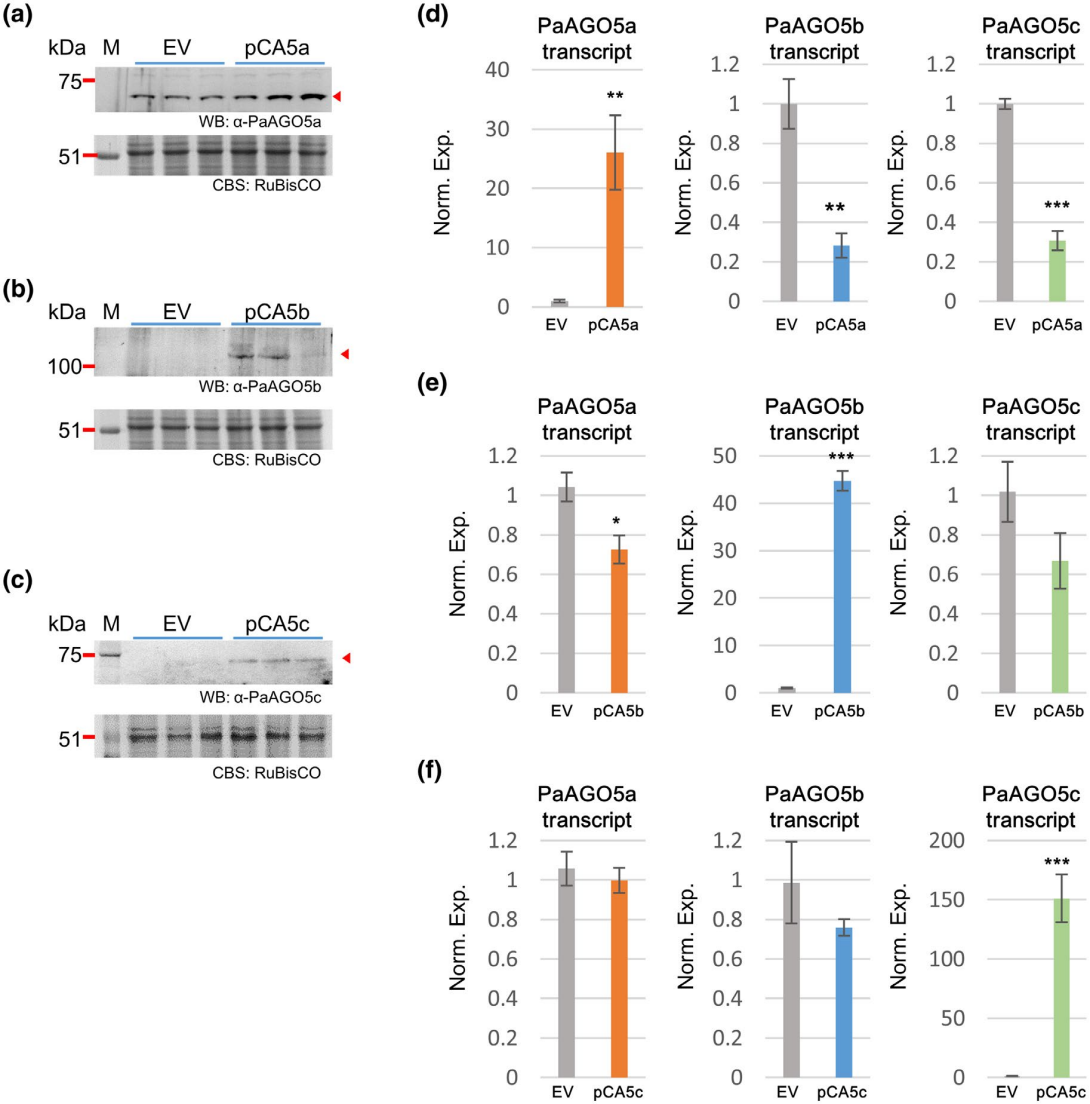
Overexpression of PaAGO5s in uninfected *Phalaenopsis aphrodite* subsp. *formosana* leaves. The constructs pCA5a, pCA5b, and pCA5c, for overexpression PaAGO5a, PaAGO5b, and PaAGO5c, respectively, were delivered into *P. aphrodite* subsp. *formosana* leaves via agroinfiltration. Leaf samples were collected at 3 days postinoculation for total protein and RNA extraction. The accumulation levels of proteins and transcripts of PaAGO5a (a,d), PaAGO5b (b,e), and PaAGO5c (c,f) were examined by using western blot (WB) and quantitative reverse transcription PCR (RT‐qPCR), respectively. The identities of samples are as indicated at the top of the lanes (for WB) or at the bottom of each column (for RT‐qPCR). The positions of viral coat proteins are indicated by the red arrowheads. M, molecular weight marker; EV, empty vector pCambia‐Ubi‐ZsGFP

**FIGURE 5:**
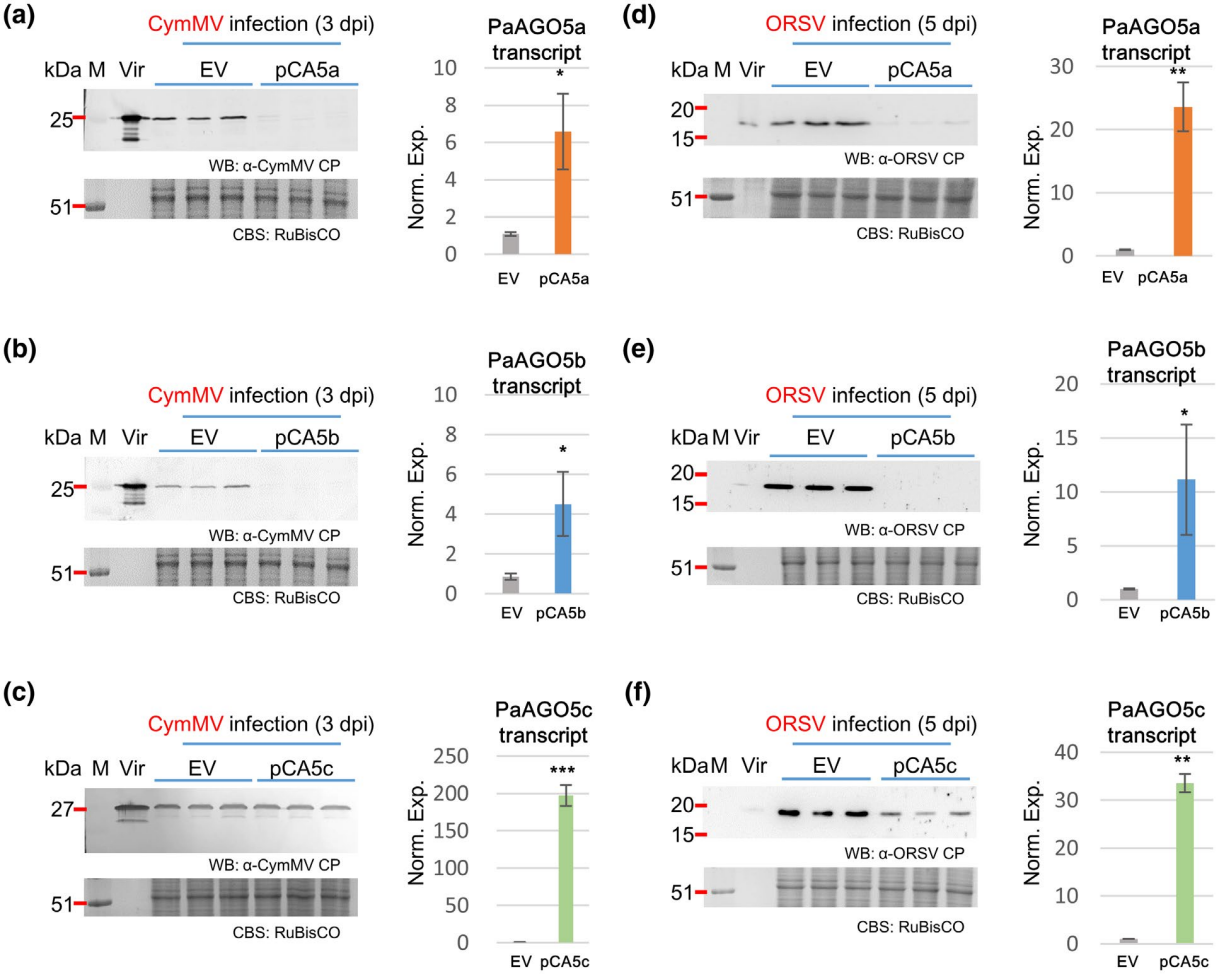
Overexpression of PaAGO5s in leaves agroinoculated with CymMV and ORSV. *Phalaenopsis aphrodite* subsp. *formosana* leaves were coinfiltrated with CymMV or ORSV infectious clones, pKCy1 or pKOR15‐2, plus pCA5a (a,d), pCA5b (b,e), or pCA5c (c,f) overexpression constructs and collected at 3 or 5 days postinoculation (dpi), respectively. Total proteins and RNA were extracted from the leaves and the accumulation level of respective PaAGO5 transcripts and the coat proteins (CPs) of CymMV and ORSV were examined by quantitative reverse transcription PCR and western blotting (WB), respectively. See legends to Figure 2 for details

To further investigate the antiviral activity, PaAGO5s were transiently overexpressed in virus‐infected plants. The overexpression of PaAGO5s did not significantly affect the antiviral activity against CymMV or ORSV infections in *N. benthamiana*, possibly due to the incompatibility of exogenous PaAGO5s and the antiviral RNA silencing machinery of *N. benthamiana*. Therefore, the antiviral activities of PaAGO5s were directly examined in *P. aphrodite* subsp. *formosana* leaves. Individual PaAGO5 coding sequences were cloned into the pCambia‐UbI1‐ZsGFP vector (Wang & Li, 2017) to generate plasmids pCA5a, pCA5b, and pCA5c for overexpression PaAGO5a, PaAGO5b, and PaAGO5c, respectively. Each plasmid was then introduced into *Agrobacterium tumefaciens* EHA105 by electroporation. The transformed *A. tumefaciens* cultures were infiltrated into *P. aphrodite* subsp. *formosana* leaves using the AGROBEST method (Wu et al., 2014). The expression of PaAGO5 transcripts was examined by RT‐qPCR and western blot. The results showed that the accumulation of PaAGO5a transcript was increased by 26.0‐fold in the leaves agroinfiltrated with pCA5a and the PaAGO5a protein could be detected (Figure 4a,d). Furthermore, the overexpression of PaAGO5a greatly suppressed the transcription levels of PaAGO5b and PaAGO5c (Figure 4d, middle and right panels). In the leaves agroinfiltrated with pCA5b or pCA5c, the accumulation levels of PaAGO5b or PaAGO5c transcripts were increased by 44.7‐ and 151.0‐fold, respectively. The production of the corresponding proteins could be readily detected (Figure 4b,c,e,f). However, the overexpression of PaAGO5b or PaAGO5c did not affect the transcription of the other PaAGO5s (Figure 4e,f, middle and right panels). These results imply that PaAGO5a may be the dominant PaAGO5 involved in the regulation of the expression of other PaAGO5s. The orchid leaves overexpressing different PaAGO5s were further inoculated with CymMV or ORSV by agroinfiltration to examine the antiviral activity of each PaAGO5. The results showed that the overexpression of PaAGO5a and PaAGO5b both significantly reduced CymMV or ORSV accumulation in leaves (Figure 5). In contrast, PaAGO5c overexpression contributed to the inhibition of ORSV accumulation but showed no significant antiviral function against CymMV. However, the overexpression of PaAGO5a and PaAGO5b reduced the accumulation of CymMV more effectively. These observations suggest that PaAGO5a and PaAGO5b might play major roles in antiviral resistance of *P. aphrodite* subsp. *formosana*, while PaAGO5c could serve a supporting/secondary role in the process.

### 2.4 Knockdown of PaAGO5s led to increased susceptibility to CymMV and ORSV infections in *P. aphrodite* subsp. *formosana*

**FIGURE 6:**
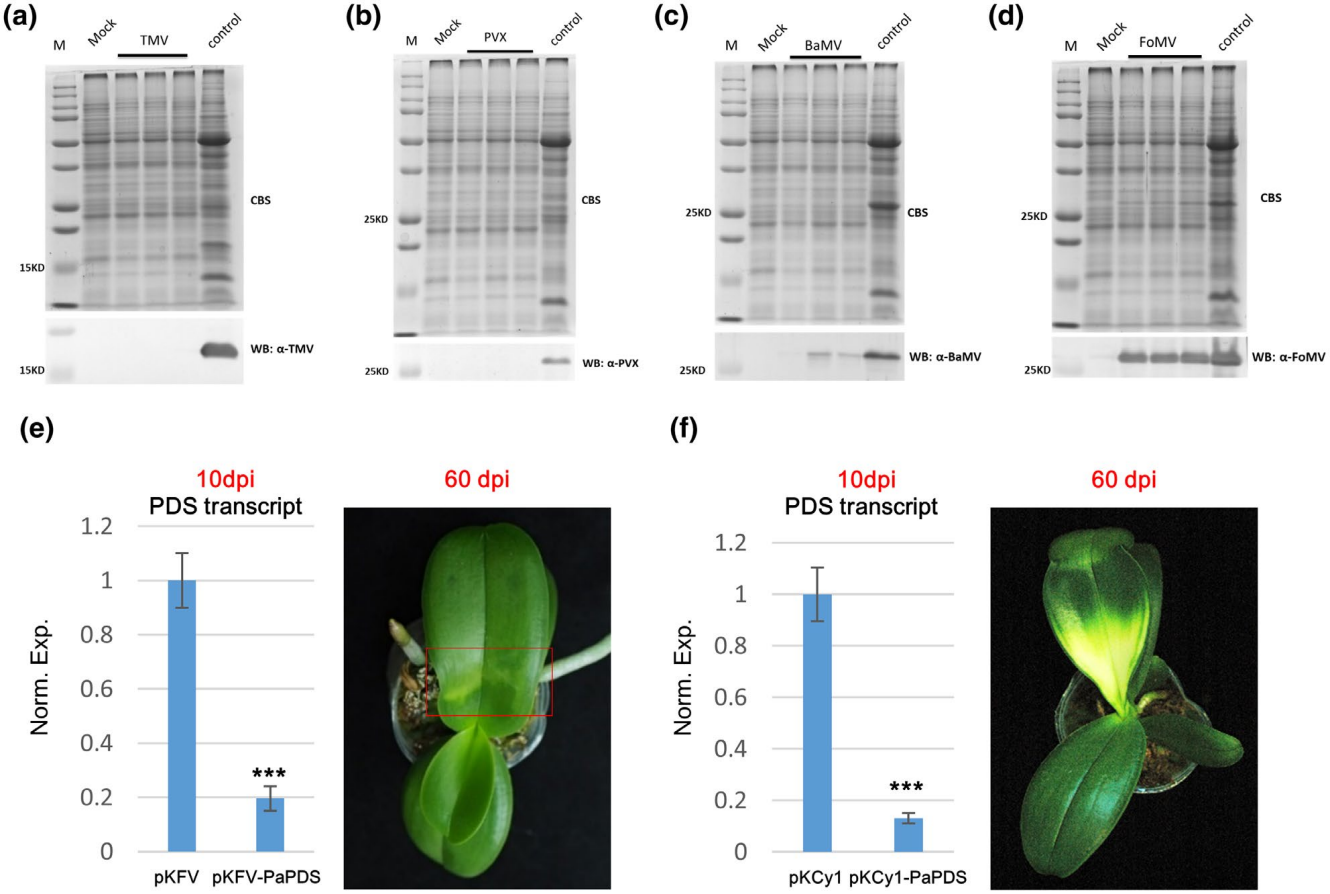
Comparison of the efficiency of virus‐induced gene silencing (VIGS) vectors in *Phalaenopsis aphrodite* subsp. *formosana* leaves. Leaves of *P. aphrodite* subsp. *formosana* were agroinfiltrated with *Agrobacterium tumefaciens* GV3850 harbouring infectious constructs of TMV (a), PVX, (b) BaMV (c), or FoMV (d). Leaf samples were collected at 3 days postinoculation (dpi) and the total proteins were extracted for analysis by sodium dodecyl sulphate polyacrylamide gel electrophoresis (SDS‐PAGE) and western blotting (WB) with specific antibodies as indicated. To test the efficiency of VIGS, *P. aphrodite* subsp. *formosana* leaves were agroinfiltrated with *A. tumefaciens* EHA105 harbouring CymMV‐ or FoMV‐based VIGS constructs, pKCy1 and pKFV, respectively, either alone or containing a *P. aphrodite* subsp. *formosana phytoene desaturase* gene (*PaPDS*) fragment, pKCy1‐PaPDS and pKFV‐PaPDS, respectively. Leaf samples were collected at 10 dpi for RNA extraction. The *PaPDS* transcript accumulation level was examined by quantitative reverse transcription PCR (e,f, left panels). The photobleaching phenotype on the leaves was recorded at 60 dpi (e,f, right panels). The accumulation levels of *PaPDS* transcripts in the VIGS‐treatment group were normalized relative to those of the empty vector‐treatment group

**FIGURE 7:**
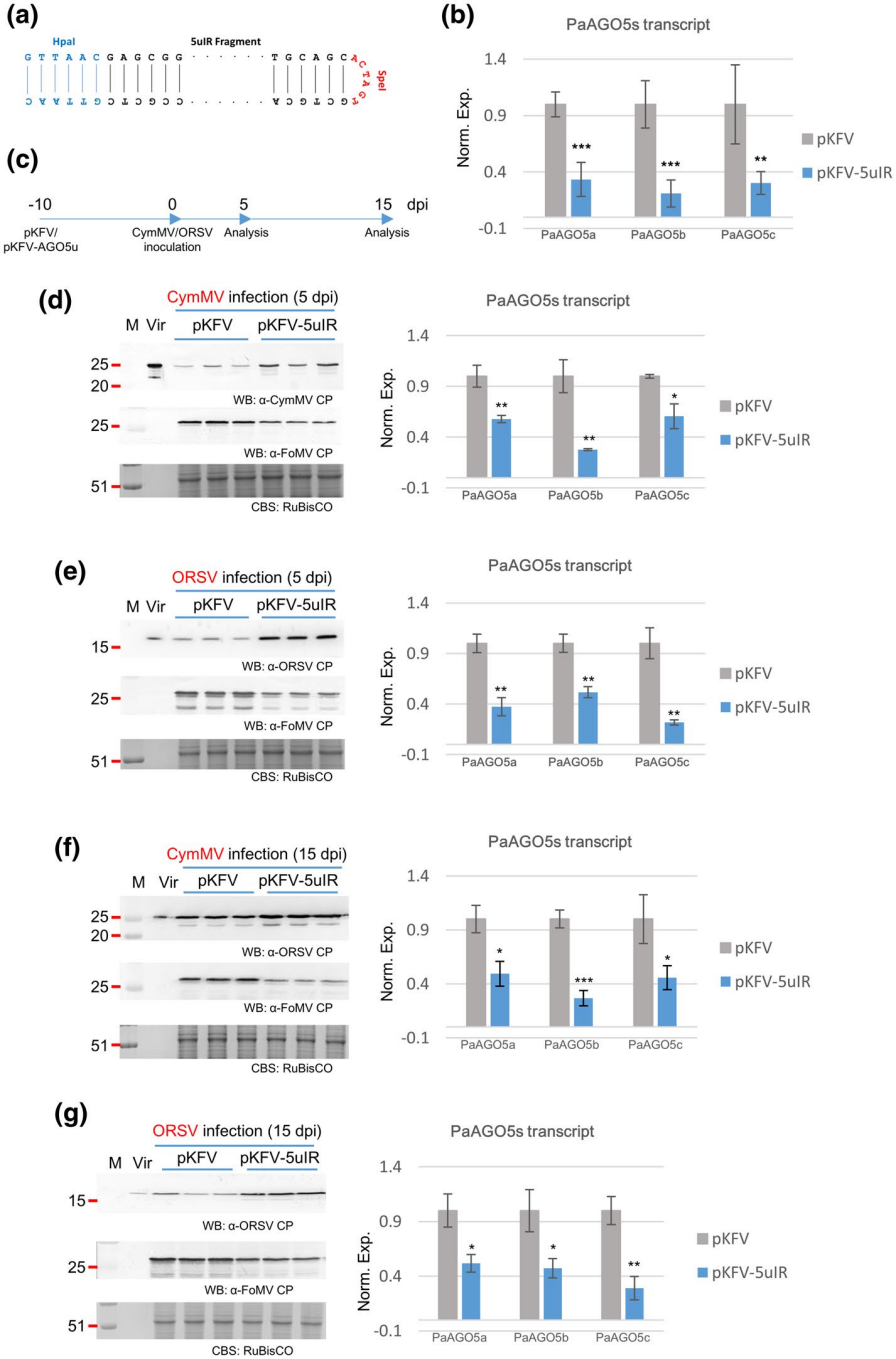
Effects of knockdown of PaAGO5s on viral accumulation. *Phalaenopsis aphrodite* subsp. *formosana* leaves were infiltrated with *Agrobacterium tumefaciens* EHA105 harbouring the virus‐induced gene silencing (VIGS) construct pKFV‐PaAGO5uIR containing a PaAGO5uIR fragment or the empty vector pKFV 10 days before (−10 days postinoculation [dpi]) the inoculation of CymMV or ORSV infectious constructs (0 dpi). Leaf samples were collected at 5 and 15 dpi. Total protein and RNA were extracted from inoculated leaves and the accumulation levels were analysed by western blotting (WB) and quantitative reverse transcription PCR (RT‐qPCR). (a) Schematic representation of PaAGO5uIR fragment. (b) Accumulation levels of PaAGO5s determined by RT‐qPCR. Leaf samples were collected at 10 dpi before viral inoculation and the accumulation levels of transcripts of PaAGO5a, PaAGO5b, and PaAGO5c were examined by RT‐qPCR as indicated. (c) Schedule for inoculation and sample collection. The accumulation levels of protein (left panels) and RNA (right panels) of CymMV and ORSV at 5 (d,e) and 15 dpi (f,g) are presented. See legends to Figure 2 for details

To further corroborate the above observations, *PaAGO5* transcripts were selectively silenced in *P*. *a* *phrodite* subsp. *formosana*, and the plants were then tested for susceptibility to CymMV or ORSV. Virus‐induced gene silencing (VIGS) is one of the most efficient tools to knock down gene expression, as long as the plant can be inoculated by the chosen viral vector. As one of the most notorious viruses on orchids, CymMV has been used to establish an effective VIGS system for studies on flowering‐related genes or plant defence response in *Phalaenopsis* orchid (Hsieh et al., 2013; Lu et al., 2012). However, CymMV‐based VIGS vectors are not applicable in this study because CymMV is the target virus under investigation. We therefore developed an alternative VIGS system for this study. Several candidate VIGS vectors were tested for infectivity on *P. aphrodite* subsp. *formosana*. The coat proteins of tobacco mosaic virus (TMV) and potato virus X (PVX) were barely detectable on leaves that had been agroinfiltrated with pKTMV and pKPVXGFP (Huang et al., 2019) at 10 dpi (Figure 6a,b). In contrast, both BaMV‐ and foxtail mosaic virus (FoMV)‐based VIGS vectors, pKBG (Prasanth et al., 2011) and pKFV, respectively, could infect *P. aphrodite* subsp. *formosana*, and the coat protein accumulation of FoMV was much higher than that of BaMV (Figure 6c,d). Accordingly, pKFV was employed as a VIGS vector for the following experiments in this study. To evaluate the silencing efficiency of the FoMV‐based VIGS vector, a fragment of the *P. aphrodite* phytoene desaturase gene (*PaPDS*) (Lu et al., 2007) was cloned into pKFV to generate pKFV‐PaPDS. The infiltration of pKFV‐PaPDS resulted in a 20% reduction of *PaPDS* transcript accumulation at 10 dpi, and the photobleaching phenotype on the inoculated leaves was observed at 60 dpi (Figure 6e). Although the photobleaching caused by FoMV‐based VIGS was not as severe as that induced by the CymMV‐based vector (Figure 6f), the accumulation of *Pa* *PDS* transcripts was reduced by either system with comparable efficiency (Figure 6e,f), demonstrating the applicability of pKFV as an effective VIGS vector in *P. aphrodite* subsp. *formosana*. To select suitable regions on respective *PaAGO5*s with high silencing efficiency and minimum off‐target effect on homologous genes (Zhou & Zeng, 2017), we performed multiple sequence alignment of the *PaAGO5* genes. However, it was found that *PaAGO5*s shared high sequence identities with each other and we were unable to identify a specific VIGS target sequence for an individual *PaAGO5* without affecting the others. The coding sequence of the PaAGO5b N‐terminal domain was more divergent than the other regions, but a sequence selected from this region failed to induce effective silencing of *PaAGO5b* expression (data not shown). Eventually, a region, PaAGO5u, located within the coding sequence of PIWI domain, conserved in the PaAGO5 clade but divergent in the other PaAGOs, was selected for silencing of all *PaAGO5*s. Following amplification by PCR with specific primer pairs (Table 1), the PaAGO5u region was cloned into pKFV as an inverted repeat, which is reported to enhance gene silencing (Smith et al., 2000; Wesley et al., 2001), to generate pKFV_5uIR (Figure 7a), which was then delivered into *P. aphrodite* subsp. *formosana* leaves through agroinfiltration. The leaves were collected and tested for PaAGO5 silencing efficiency at 10 dpi. The results showed that the transcripts of PaAGO5a, PaAGO5b, and PaAGO5c were decreased to 33%, 21%, and 30%, respectively, as compared to the empty vector‐treated group (Figure 7b). Although the transcript level of PaAGO5b was slightly increased following FoMV infection (Figure S3), all the PaAGO5 transcripts were knocked down via the infiltration of the pKFV_5uIR vector. Next, at 10 days after infiltration of pKFV_5uIR, the PaAGO5‐silenced leaves were further challenged with CymMV or ORSV (Figure 7c, 0 dpi). Leaf samples were collected after 5 or 15 days (Figure 7c, 5 or 15 dpi) for viral accumulation analysis (Figure 7c). The results revealed that silencing of PaAGO5s increased the accumulation of CymMV and ORSV at 5 dpi (Figure 7d,e). Although the accumulation of CymMV showed no significant difference at 15 dpi in different groups, the ORSV accumulation remained higher in PaAGO5s‐silenced leaves (Figure 7f,g). The accumulation of FoMV was shown to be hampered by CymMV or ORSV challenge; nevertheless, all of the PaAGO5s were significantly silenced by the pKFV_5uIR VIGS vector throughout all treatments (Figure 7, right panel). Together, the results indicate that PaAGO5s are the key elements in the defence mechanism against CymMV and/or ORSV in *P. aphrodite* subsp. *formosana*.

## 3 DISCUSSION

### 3.1 PaAGO5a and PaAGO5b play central roles in antiviral defence in *P. aphrodite* subsp. *formosana*

AGO proteins are known to be essential components in the RNA‐induced silencing complex (RISC) involved in the RNA interference (RNAi) mechanisms of many organisms. AGO proteins are responsible for the interaction with guide RNAs, translational inhibition, and/or cleavage of the target RNAs (Carbonell & Carrington, 2015; Fang & Qi, 2016; Zhang et al., 2015). It has been shown that different sets of AGO proteins may participate in the regulation of unique biological functions in different organisms or within the same organism (Brosseau & Moffett, 2015). The AGO proteins involved in antiviral defences in several model plants have been extensively studied, but relatively little was known for the AGOs responsible for resistance against viruses in orchids previously. In this study, we identified all 11 AGO proteins in *P. aphrodite* subsp. *formosana* and analysed their accumulation levels following virus infections. We further verified the antiviral functions of specific PaAGO5s through transient gene overexpression and knockdown approaches. The results revealed that two members of the PaAGO5 family proteins, PaAGO5a and PaAGO5b, play key roles in the antiviral machineries.

To further analyse the effects of other PaAGOs in the defence against CymMV infection, VIGS assays were performed for individual PaAGOs. Except for PaAGO7a, pKFV successfully knocked down the gene expression of PaAGO1a/b, PaAGO4, PaAGO6, PaAGO7b, PaAGO10a, and PaAGO10b (Table S1; Figure S5). The results of the CymMV inoculation assay discovered that silencing of PaAGO1a/b and PaAGO10a significantly increased CymMV accumulation by 2.0‐ and 1.8‐fold, respectively (Figure S5a,b,k,l), similar to that caused by the silencing of PaAGO5s, 1.4‐fold (Figure S6), while silencing of AGO10b had no significant effect on CymMV RNA accumulation. The results indicate that the other members of the AGO1/10 clade may also participate in orchid antiviral defences, although the expression levels of these members were not elevated significantly in response to CymMV infections. Nevertheless, this study revealed that PaAGO5b is an important antiviral protein that may serve as the frontline defence against CymMV and/or ORSV infections at the early stage, as PaAGO5b is the only significantly activated gene upon viral infections.

### 3.2 Other members of the AGO1/5/10 clade are also reactive to viral infections

In addition to PaAGO5b, the expression of other genes in the AGO1/5/10 clade, PaAGO1a and PaAGO10s, were also found to be responsive to CymMV and/or ORSV infections in this study. AGO1 is widely and constitutively expressed in many model plants and is responsible for the regulation of various genes through loading different microRNAs (miRNAs). The miR168‐loaded AGO1 decreases AGO1 transcription and maintains AGO1 protein homeostasis. Viral infections can induce a higher level of miR168, which results in the reduction of AGO1 protein and antiviral activity (Lang et al., 2011; Várallyay et al., 2010). *P. aphrodite* subsp. *formosana* encodes two orthologues of AGO1, PaAGO1a and PaAGO1b. The expression of PaAGO1a was twice that of PaAGO1b in healthy orchids, implying that PaAGO1a may also play a predominant role in orchid. Furthermore, ORSV infection significantly decreased the expression of PaAGO1a and, to a lesser extent, PaAGO1b. However, CymMV or mixed infection did not affect the expression of both PaAGO1 transcripts. The observations suggested that ORSV was able to suppress the accumulation of PaAGO1b, possibly through manipulating the expression of miR168 in *P. aphrodite* subsp. *formosana*. Another member of the AGO1/5/10 clade, AGO10, is also known to regulate several physiological characteristics in plants through competing with AGO1 for specific miRNAs. For instance, AGO10 has been proposed to act as a decoy to sequestrate miR165/166 from loading into AGO1 and regulate the maintenance of the shoot apical meristem (Roodbarkelari et al., 2015; Zhu et al., 2011). Furthermore, NbAGO10 enhances the accumulation of BaMV by sequestering and degrading BaMV‐derived vsiRNAs, preventing their incorporation into NbAGO1 (Huang et al., 2019). However, in *P. aphrodite* subsp. *formosana,* PaAGO10a and PaAGO10b were both significantly decreased while singly or doubly infected with CymMV and/or ORSV (Figure 3e). Our observations suggest that CymMV and ORSV may have evolved the ability to suppress the antiviral defences mediated by PaAGO1s and/or PaAGO10s in *P. aphrodite* subsp. *formosana*, which in turn may have developed the ability to employ PaAGO5s as the primary antiviral components.

### 3.3 The asymmetric synergism between CymMV and ORSV might be related to the differential responses of PaAGOs

Similar to previous reports (Pai et al., 2020), asymmetric synergism between CymMV and ORSV was also observed in this study, in which CymMV is the primary beneficiary in the synergistic relationship. The coat protein accumulation of CymMV was significantly higher under mixed‐infection conditions at 15 dpi only; however, the accumulation of ORSV coat protein was significantly suppressed throughout the experiment (Figure 2). Although it could not be ruled out that CymMV might simply deprive ORSV of certain factors for replication or accumulation, it is likely that other viral or host factors are also involved in the asymmetric synergism. Potexviruses, such as PVX and BaMV, encode TGBp1 proteins (p25 and p28, respectively) that are reported to be the important viral suppressors of RNA silencing (VSRs) for viral infections (Aguilar et al., 2015; Hsu et al., 2004). Moreover, p25 has been reported to inhibit the systemic movement of silencing signals in *N. benthamiana* (Voinnet et al., 2000). AGOs may also be involved in the process. AGO1 and AGO7 were found to be destabilized by PVX p25 (Brosseau & Moffett, 2015). AGO1, AGO6, AGO7, AGO9, and AGO10 exhibited lower expression levels with the coexpression of Plantago asiatica mosaic virus p25 (Brosseau et al., 2016). In contrast, members of the genus *Tobamovirus*, such as TMV and ORSV, also encode a well‐studied VSR, p126. TMV p126 consists of three domains, methyltransferase, helicase, and nonconserved regions, and each of them has been reported to function independently as a silencing suppressor (Wang et al., 2012). A previous study showed that TMV p126 could increase the susceptibility of *N. benthamiana* to alfalfa mosaic virus, brome mosaic virus, TRV, and PVX infections (Harries et al., 2009). These studies demonstrated how VSRs help the corresponding viruses thwart the attacks of plant defence systems, and even improve the infection of other viruses. In the present study, PaAGO4, PaAGO6, PaAGO7s, and PaAGO10s were all down‐regulated in all virus‐infected groups (Figure 3). However, the accumulation of PaAGO5b was significantly elevated when *P. aphrodite* subsp. *formosana* plants were infected singly with CymMV or doubly with both viruses, but not ORSV alone. Although there was no direct evidence demonstrating that CymMV TGBp1 and/or ORSV p126 were involved in the suppression of RNA silencing, CymMV and ORSV may encode effective VSRs that alter the host transcriptome and regulate the expression of certain host proteins. The identification of the VSRs encoded by CymMV and ORSV, and the investigation of VSR‐associated miRNAs or vsiRNAs may shed light on the regulation of PaAGOs during viral infection and the synergism mechanisms between CymMV and ORSV.

### 3.4 PaAGO5s harbour unique features in addition to the conserved domains

In addition to the PAZ, MID, and PIWI domains conserved among AGO proteins, PaAGO5b contained a variable N‐terminal domain. Previous studies suggested that the N‐terminal domain possesses activity in the stabilization of ternary interactions in AGO complex, unwinding sRNA duplex, and targeting RNA cleavage (Hauptmann et al., 2013; Kwak & Tomari, 2012; Wang et al., 2009). It is therefore possible that the N‐terminal domain also participates in posttranscriptional gene silencing through versatile mechanisms, but might be dispensable for the general functions of AGO proteins. PaAGO5a mRNA contains a long 5′ untranslated region of 453 nt in length where a putative upstream open reading frame (uORF) was found that might regulate the translation of PaAGO5a. Such uORFs frequently interfer with unrestrained ribosomal scanning toward the main protein initiation codon (Wilkie et al., 2003). PaAGO5a did not respond to viral infection at an early stage of viral infection, but the amount of PaAGO5a protein was up‐regulated in the leaves infected by CymMV or by CymMV in combination with ORSV at 20 dpi (Figure S4). The overexpression of PaAGO5a was able to decrease the expression of PaAGO5b and PaAGO5c, and also provide antiviral activity against both viruses (Figures 4d and 5a,d). *A. thaliana* AGO5 has been speculated as the second layer of defence in the absence of AGO2 (Brosseau & Moffett, 2015; Garcia‐Ruiz et al., 2015). Generally, all the PaAGO5s exhibited varying degrees of antiviral abilities and might participate in multilayer resistance in orchid against viruses. The unique features on PaAGO5 proteins or mRNAs might have participated in the regulation of the antiviral defence mechanisms.

### 3.5 A hypothetical model for the role of PaAGO5s in the antiviral defence of *P. aphrodite* subsp. *formosana*

**FIGURE 8:**
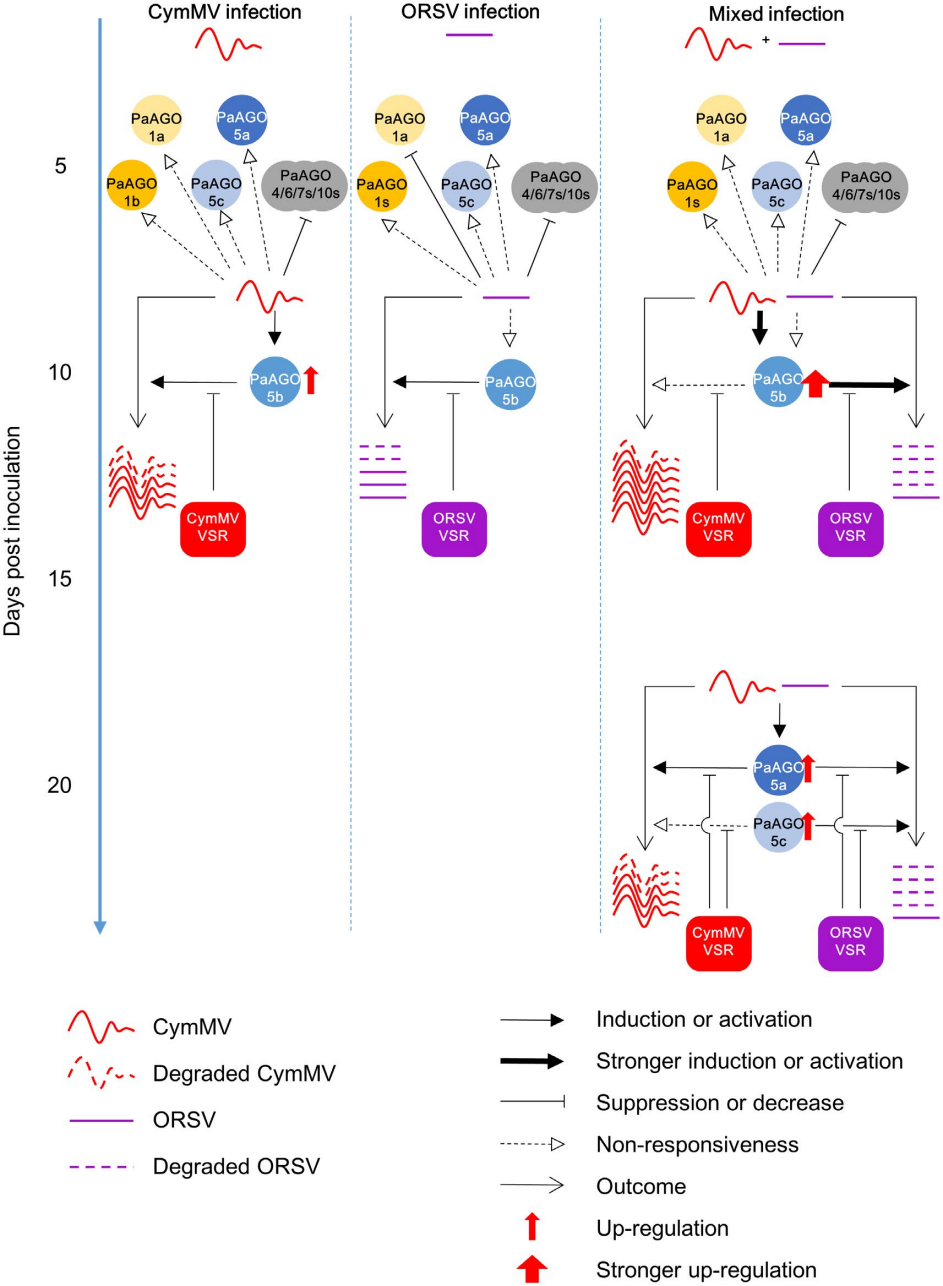
A proposed model illustrating the correlation between the expression of PaAGOs and the accumulation of CymMV and/or ORSV. The arrow, blunt‐ended line, and dashed arrow symbolize induction, suppression, and nonresponsiveness of the interactions, respectively. Increased accumulation levels of AGO proteins are symbolized by the red upward arrows. The thickness of the arrows indicates different levels of enhancement, the thicker the arrow, the greater the enhancement. See main text for details

For better comprehension, we propose a model to illustrate the correlation between PaAGOs and viral accumulation (Figure 8). Through the coevolution of *P. aphrodite* subsp. *formosana* with CymMV and ORSV, the viruses have developed VSRs that suppress the accumulation of most PaAGOs (including AGO4/6/7/10s, grey circles in Figure 8), and restrain the expression of PaAGO1s, PaAGO5a, and PaAGO5c. To counteract this, *P. aphrodite* subsp. *formosana* expresses PaAGO5b rapidly in response to the infection of CymMV (Figure 8, left), resulting in the degradation of CymMV RNA. However, in single infection conditions, ORSV VSR is still able to restrain PaAGO5b expression at the basal level (Figure 8, middle), with only minor degradation of ORSV RNA. When *P. aphrodite* subsp. *formosana* leaves are coinfected by CymMV and ORSV, the expression of PaAGO5b is greatly enhanced by the presence of both the pathogen‐associated molecular patterns of CymMV and ORSV. PaAGO5b may exhibit a binding preference to ORSV RNA, rendering ORSV RNA more vulnerable to PaAGO5b‐mediated degradation (as symbolized by the thicker arrow signs). The diverted binding of PaAGO5b to ORSV RNA may thereby allow CymMV to evade the surveillance system (as symbolized by the dashed arrow for nonresponsiveness of PaAGO5b interference), resulting in the asymmetrical synergism in which CymMV accumulation increases while that of ORSV decreases in the mixed‐infection condition (Figure 8, right). Overexpression of PaAGO5a or PaAGO5b at an early stage of infection (3 dpi) hampers CymMV replication, probably due to the relative underexpression of VSR at the early stage. The slight up‐regulation of PaAGO5a and PaAGO5c in mixed infection at a later stage (c.20 dpi) implies that they may function as a second layer of the plant immune system (Figure 8, lower right, and Figure S4). PaAGO5b expression might be slightly induced by the infiltration of the FoMV‐based vector, pKFV, and slightly delay the replication of CymMV infection (Figure S3). However, silencing of PaAGO5s significantly disrupts the plant resistance, leading to a higher accumulation level of CymMV and ORSV.

### 3.6 Conclusion

This study identified and characterized the expression of all 11 AGO proteins in *P. aphrodite* subsp. *formosana* under viral infections for the first time and revealed that PaAGO5s are the key players in the antiviral defence mechanisms. Further overexpression and gene silencing experiments verified the functions of PaAGO5s against CymMV and ORSV. The results provided deeper insights into the AGO‐related antiviral mechanism in *P. aphrodite* subsp. *formosana,* and suggested a promising strategy to develop resistance against viruses in orchid, either through breeding or transgenic approaches to enhance the expression of PaAGO5s. Future studies will focus on the regulation of PaAGO5 expressions in response to viral infections to decipher the interaction between viral factors and PaAGO5‐associated resistance in *P. aphrodite* subsp. *formosana*.

## 4 EXPERIMENTAL PROCEDURES

### 4.1 RNA extraction, RT‐PCR, and quantitative PCR

Total RNA extraction and the elimination of DNA contamination were performed by using Direct‐zol RNA MiniPrep (Zymo Research). Following extraction, 2 µg of total RNA was used for first‐strand cDNA synthesis with oligo(dT_18_) primer and GoScript Reverse Transcriptase (Promega). The first‐strand cDNA was then mixed with specific primer and KAPA SYBR FAST qPCR master mix (Kapa Biosystems). Quantitative real‐time PCR was carried out using a TOptical Gradient 96 Real‐Time PCR thermal cycler (Biometra) as described (Huang et al., 2019). The primer sequences are as listed in Table 1. Expression levels of target transcripts were normalized to the geometric mean of housekeeping gene, *Actin*, to control the variability and further analysed using the 2^−ΔΔ^ *^C^* ^t^ method (Livak & Schmittgen, 2001). For the confirmation of reproducibility, three biological replicates of each essay were used for qPCR analysis, and three technical replicates were analysed for each biological replicate.

### 4.2 Construction of PaAGO5 overexpression vector

The PaAGO5a, PaAGO5b, and PaAGO5c coding sequences (cds) were amplified by RT‐PCR. The specific primer pairs were designed according to the cds of PaAGO5s in the Orchidstra 2.0 database as listed in Table 1. Each PCR amplicon contains a FLAG tag sequence at the 5′ end and is flanked by *Kpn*I and *Sac*I restriction sites. The purified PCR products and pEpyon‐32K plasmid (Chen et al., 2011) were digested using *Kpn*I and *Sac*I, and ligated by T4 DNA ligase at 16 °C for 12 hr to generate expression constructs pEpA5a, pEpA5b, and pEpA5c. To construct the vector for PaAGO5 overexpression in *P. aphrodite* subsp. *formosana*, pCAMBIA‐Ubi1‐ZsGFP (Wang & Li, 2017) was digested with *Mlu*I, followed by an end repair with T4 DNA polymerase to generate the linear form of plasmid DNA containing blunt ends. Then, the plasmid were further digested with *Sac*I to remove the *Zea mays* codon‐optimized *GFP* gene from the pCAMBIA vector. pEpA5a, pEpA5b, and pEpA5c were digested with *Kpn*I, followed by an end repair with T4 DNA polymerase and digestion with *Sac*I as mentioned above. The digested PaAGO5a, PaAGO5b, and PaAGO5c cds fragments and pCAMBIA vector were ligated with T4 DNA ligase to create the expression constructs pCU‐A5a, pCU‐A5b, and pCU‐A5c, respectively.

### 4.3 Construction of pKCy1, pKOR15‐2, pKTMV, and FoMV‐based VIGS vector, pKFV

CymMV genomic DNA was obtained from infectious clone pCCy1 (Lee et al., unpublished) by *Sbf*I and *Xho*I digestion and further ligated with *Sbf*I/*Xho*I‐digested pKn vector (Liou et al., 2014) to generate the infectious clone pKCy1. Genomic RNAs of ORSV and TMV were extracted from ORSV‐infected *P. aphrodite* subsp. *formosana* leaves and TMV‐infected *N. benthamiana* leaves, respectively. The cDNAs of viral genomes were amplified by RT‐PCR with a primer pair containing flanking *Hin*dIII and *Sac*I sites and ligated with *Hin*dIII/*Sac*I‐digested pKn vector to generate infectious clones pKOR1 and pKTMV, respectively. The FoMV‐based VIGS cassette was obtained from pCFV (Huang et al., 2020) by *Sbf*I and *Xho*I digestion and then ligated with *Sbf*I/*Xho*I‐digested pKn vector to generate the pKFV vector. The fragment for gene silencing of *P. aphrodite* subsp. *formosana* endogenous phytoene desaturase (*PaPDS*) was amplified by PCR using *PDS* primers PDS‐F and PDS‐R (Lu et al., 2007). The fragment of *PaPDS* flanked by *Age*I and *Not*I restriction sites was cloned into VIGS vector pKCy1 to generate the pKCy1‐PDS vector. The pKFV‐PDS VIGS construct was created similarly using *Hpa*I‐ and *Mlu*I‐digested *PaPDS* fragment and pKFV vector. The fragment used for silencing all three *PaAGO5* genes, PaAGO5 u, in *P. aphrodite* subsp. *formosana* was flanked by *Hpa*I and *Spe*I at the 5′ and 3′ ends, respectively. After PCR amplification, the product was digested by *Spe*I and self‐ligated to generate an inverted repeat fragment of PaAGO5 u, designated PaAGO5uIR. PaPDS and PaAGO5uIR fragments were digested by *Hpa*I and ligated with *Hpa*I‐digested pKFV to generate pKFV‐PaPDS and pKFV‐PaAGO5uIR, respectively.

### 4.4 Agroinfiltrations and virus inoculations in *P. aphrodite* subsp. *formosana*

For the agroinfiltration on *P. aphrodite* subsp. *formosana* leaves, the AGROBEST method (Wu et al., 2014) with slight modification was employed. Briefly, pCAMBIA‐Ubi1‐ZsGFP, pCAMBIA‐Ubi1‐PaAGO5, pKCy1, and pKOR15‐2 constructs were electroporated into *A. tumefaciens* EHA105. Aliquots of 2 ml saturated culture of agrobacteria were poured into 18 ml ApKa Luria Bertani (LB) medium and incubated at 28 °C for 3 hr. Bacterial cells were then collected by centrifugation and incubated in AB‐MES buffer under constant shaking (60 rpm) at 28 °C for 24 hr, collected, and resuspended in AB‐MES + 1/2 × MS infiltration buffer containing 200 µM acetosyringone. The suspension of agrobacteria containing expression constructs was adjusted to give an OD_600_ of 10. The suspension of agrobacteria containing infectious clones pKCy1 and pKOR15‐2 was adjusted to an OD_600_ of 0.5 to 1.5.

### 4.5 Protein analysis

*P. aphrodite* subsp. *formosana* leaves were ground to powder with liquid nitrogen and approximately 0.2 g leaf powder was soaked in 250 µl extraction buffer (20 mM Tris‐HCl, pH 7.5, 5 mM MgCl_2_, 150 mM NaCl, 5 mM dithiothreitol, 0.5% NP‐40) amended with 1% protease inhibitor cocktail (Roche). The extracted proteins were analysed by sodium dodecyl sulphate polyacrylamide gel electrophoresis (SDS‐PAGE) and western blot (Burnette, 1981; Laemmli, 1970).

## Supporting information


**FIGURE S1** The transcript expression profile of PaAGOs in different tissues. PaAGO transcript expression (FPKM and TPM) was adapted from the Orchidstra 2.0 database. The expressions of different PaAGOs in leaves (a) and different tissues (b) are shown

**FIGURE S2** The functional domain(s) of PaAGO5 proteins. (a) Schematic representation of PaAGO5 protein domains. (b) Alignment of the amino acid sequences of *Arabidopsis thaliana* AGO1, 5, and 10 and PaAGO5s. The positions of the DDH catalytic triad (blue arrow heads) and small RNA interacting region (red boxes) are indicated

**FIGURE S3** The PaAGO5b transcript expression in the leaves inoculated with CymMV or FoMV. *Phalaenopsis aphrodite* subsp. *formosana* leaves were inoculated with CymMV or ORSV and collected at 10 days postinoculation for RNA extraction. The accumulation of PaAGO5b transcript (left panels) was assayed by quantitative reverse transcription PCR. The leaves were agroinfiltrated with *Agrobacterium tumefaciens* EHA105 harbouring pKCy1 (CymMV) and pKFV (FoMV), respectively, or the empty vector (EV). Values are means ± *SD* of three biological replicates. Norm. Exp, normalized expression level; *, **, and ***, significant difference at *p* < .05, *p* < .01, and *p* < .001 determined by Student’s *t* test, respectively

**FIGURE S4** The PaAGO5s overexpression in leaves which were inoculated with CymMV and/or ORSV. *Phalaenopsis aphrodite* subsp. *formosana* leaves were inoculated with CymMV (a), ORSV (b), or mixed (c) infection of both viruses via agroinfiltration. The leaves were collected at 5, 10, 15, and 20 days postinoculation for RNA extraction. The transcript expression level of PaAGO5a (left panels), PaAGO5b (middle panels), and PaAGO5c (right panels) were assayed by quantitative reverse transcription PCR. Data sets that are significant at different levels are indicated: *p < .05, **p < .01, ***p < .001

**FIGURE S5** Effects of knockdown of PaAGO1s/4/6/7s/10s on CymMV accumulation. *Phalaenopsis aphrodite* subsp. *formosana* leaves were infiltrated with *Agrobacterium tumefaciens* EHA105 harbouring the virus‐induced gene silencing (VIGS) constructs for silencing of respective PaAGOs (Table S1, as indicated on the top of each panel) or the empty vector pKFV 10 days before (−10 days postinoculation [dpi]) the inoculation of CymMV infectious constructs (0 dpi). Leaf samples were collected at 5 dpi. Total protein and RNA were extracted from inoculated leaves and the accumulation levels of CymMV and PaAGOs were analysed by western blot and quantitative reverse transcription PCR, respectively. The CymMV coat protein accumulation (CyCP) was quantified and plotted. The results of gene silencing of PaAGO1a and 1b (a,b), PaAGO4 (c,d), PaAGO6 (e,f), PaAGO7a (g,h), PaAGO7b (i,j), PaAGO10a (k,l), and PaAGO10b (m,n) are presented. Samples from leaves agroinfiltrated with pKFV plus pKn (m1) and respective PaAGO silencing construct plus pKn (m2) were used as negative controls

**FIGURE S6** Quantitative analysis of knockdown of PaAGO5s on CymMV accumulation. To determine specifically the effect of the silencing of individual PaAGO5s on CymMV accumulation, the data were extracted from the result presented in the western blot (left panel) of Figure 7d. The CymMV coat protein (CyCP) accumulation levels were determined by quantitative band density analysis. All bands were normalized to the accumulation level of RuBisCO

**TABLE S1** List of the primers used for cloning virus‐induced gene silencing fragments

## Data Availability

The data supporting the findings of this study are available from the corresponding author upon request.
