# Supplementary figures and images for: Argonaute 5 family proteins play crucial roles in the defence against Cymbidium mosaic virus and Odontoglossum ringspot virus in Phalaenopsis aphrodite subsp. formosan a
Source: Mol Plant Pathol. 2021 Mar 21;22(6):627–43. doi: 10.1111/mpp.13049 (PMC8126185; doi:10.1111/mpp.13049)

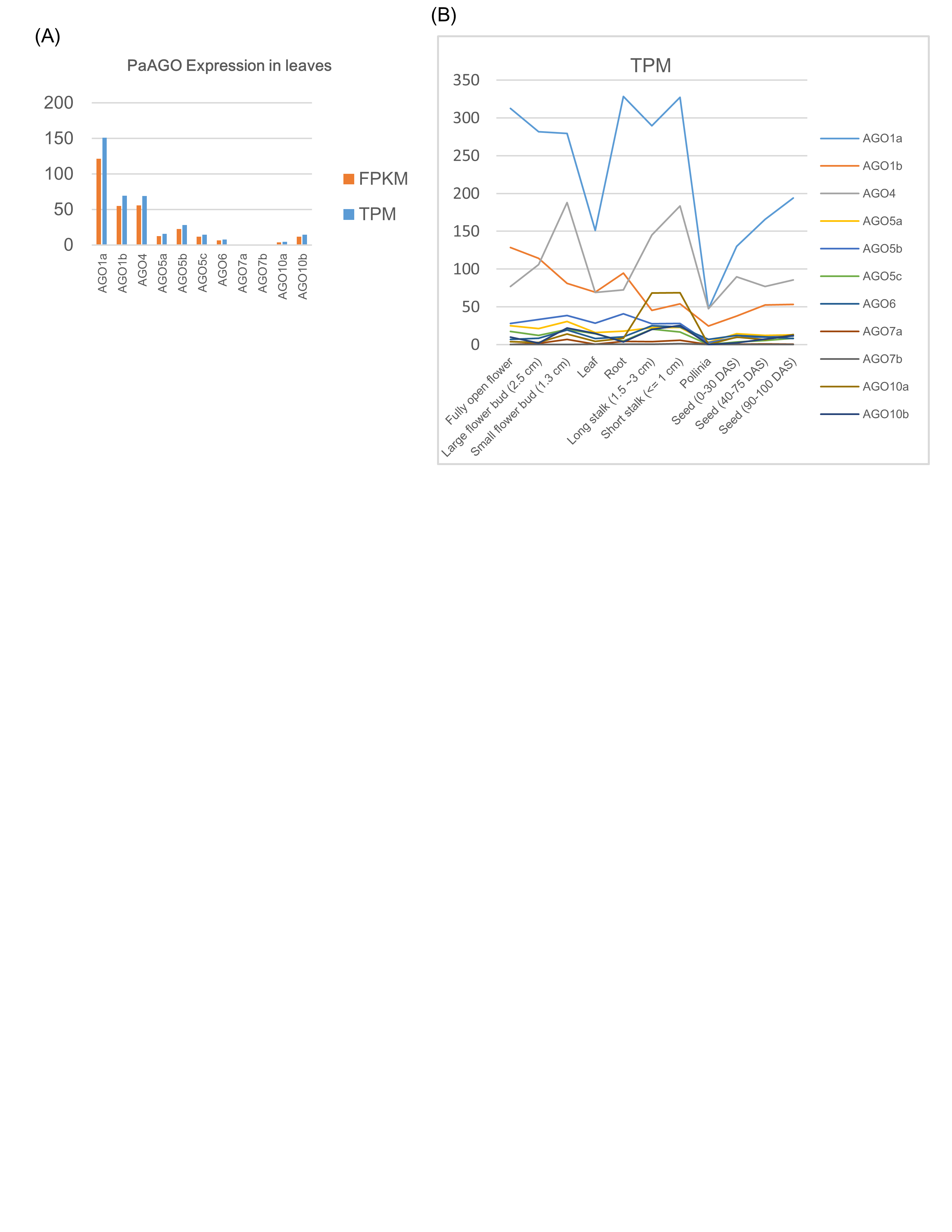

Supplement: Supplementary file 1 — FIGURE S1 The transcript expression profile of PaAGOs in different tissues. PaAGO transcript expression (FPKM and TPM) was adapted from the Orchidstra 2.0 database. The expressions of different PaAGOs in leaves (a) and different tissues (b) are shown [file MPP-22-627-s001.TIF]

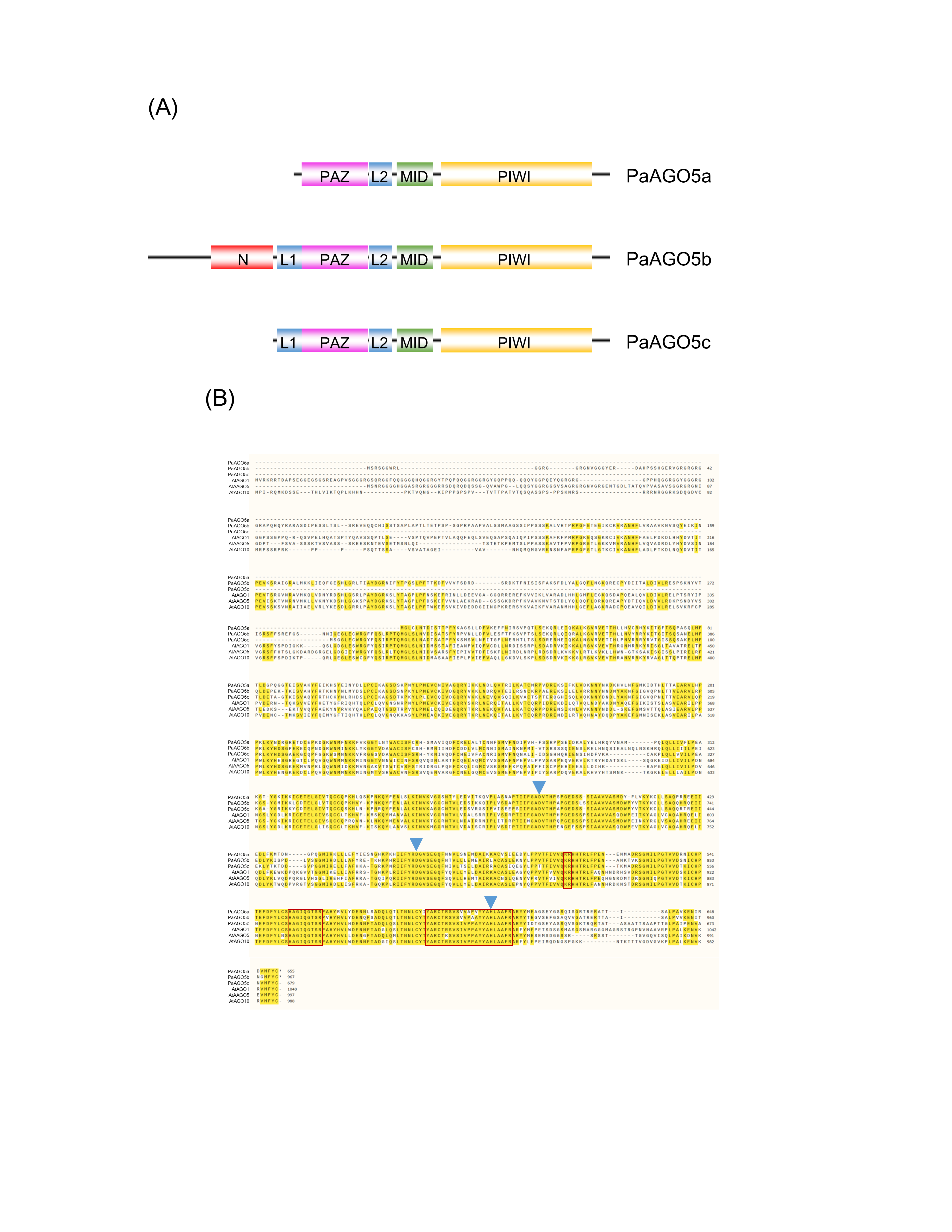

Supplement: Supplementary file 2 — FIGURE S2 The functional domain(s) of PaAGO5 proteins. (a) Schematic representation of PaAGO5 protein domains. (b) Alignment of the amino acid sequences of Arabidopsis thaliana AGO1, 5, and 10 and PaAGO5s. The positions of the DDH catalytic triad (blue arrow heads) and small RNA interacting region (red boxes) are indicated [file MPP-22-627-s007.TIF]

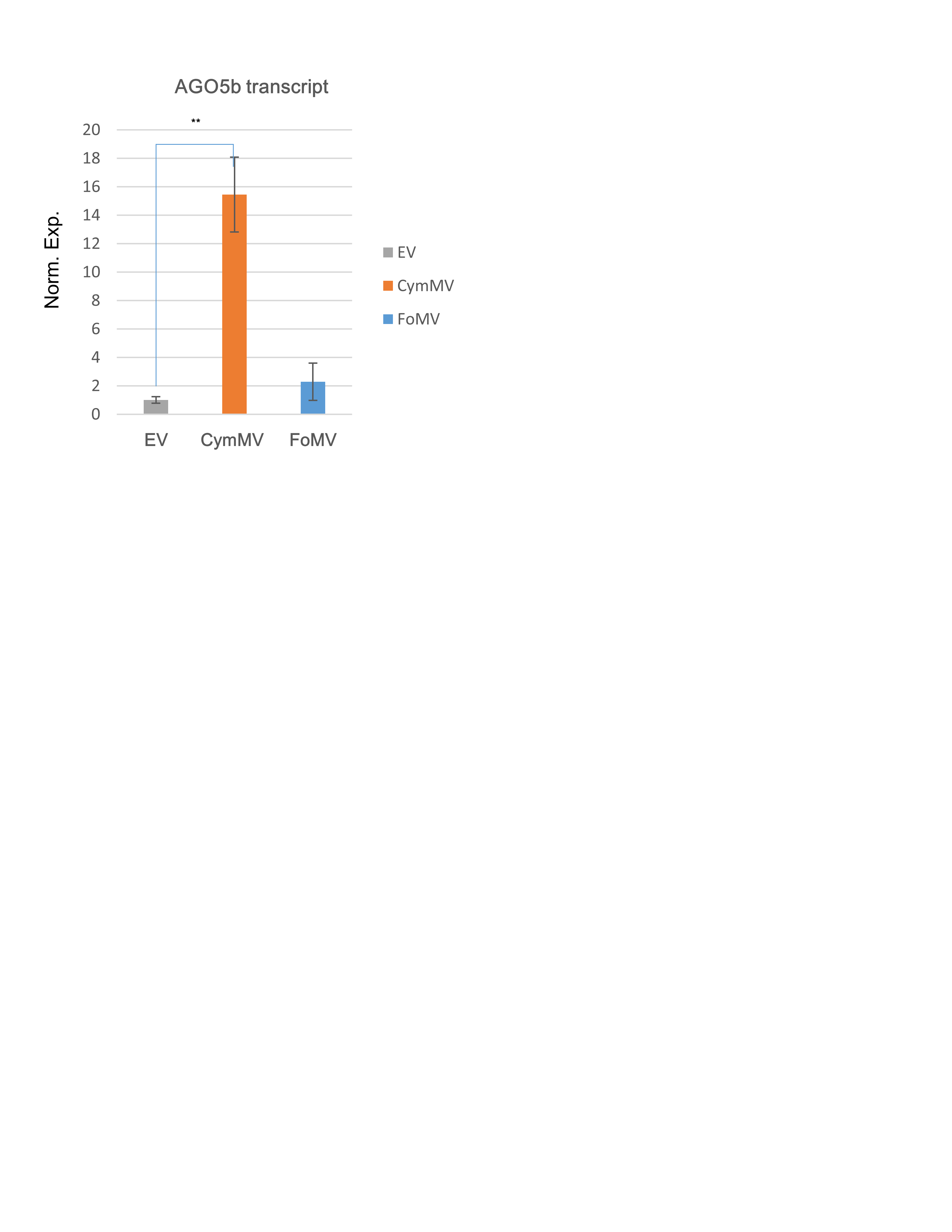

Supplement: Supplementary file 3 — FIGURE S3 The PaAGO5b transcript expression in the leaves inoculated with CymMV or FoMV. Phalaenopsis aphrodite subsp. formosana leaves were inoculated with CymMV or ORSV and collected at 10 days postinoculation for RNA extraction. The accumulation of PaAGO5b transcript (left panels) was assayed by quantitative reverse transcription PCR. The leaves were agroinfiltrated with Agrobacterium tumefaciens EHA105 harbouring pKCy1 (CymMV) and pKFV (FoMV), respectively, or the empty vector (EV). Values are means ± SD of three biological replicates. Norm. Exp, normalized expression level; *, **, and ***, significant difference at p < .05, p < .01, and p < .001 determined by Student’s t test, respectively [file MPP-22-627-s004.TIF]

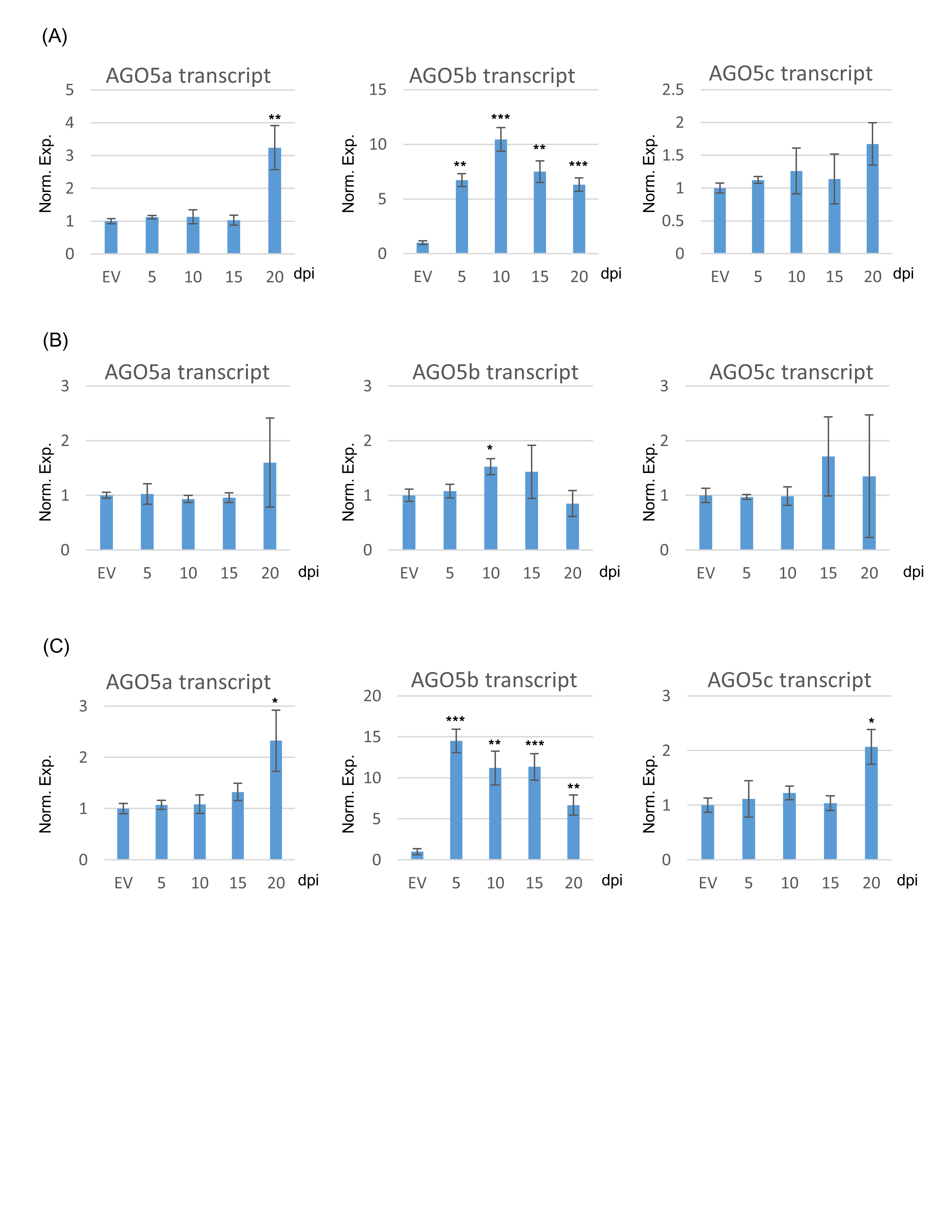

Supplement: Supplementary file 4 — FIGURE S4 The PaAGO5s overexpression in leaves which were inoculated with CymMV and/or ORSV. Phalaenopsis aphrodite subsp. formosana leaves were inoculated with CymMV (a), ORSV (b), or mixed (c) infection of both viruses via agroinfiltration. The leaves were collected at 5, 10, 15, and 20 days postinoculation for RNA extraction. The transcript expression level of PaAGO5a (left panels), PaAGO5b (middle panels), and PaAGO5c (right panels) were assayed by quantitative reverse transcription PCR. Data sets that are significant at different levels are indicated: *p < .05, **p < .01, ***p < .001 [file MPP-22-627-s005.TIF]

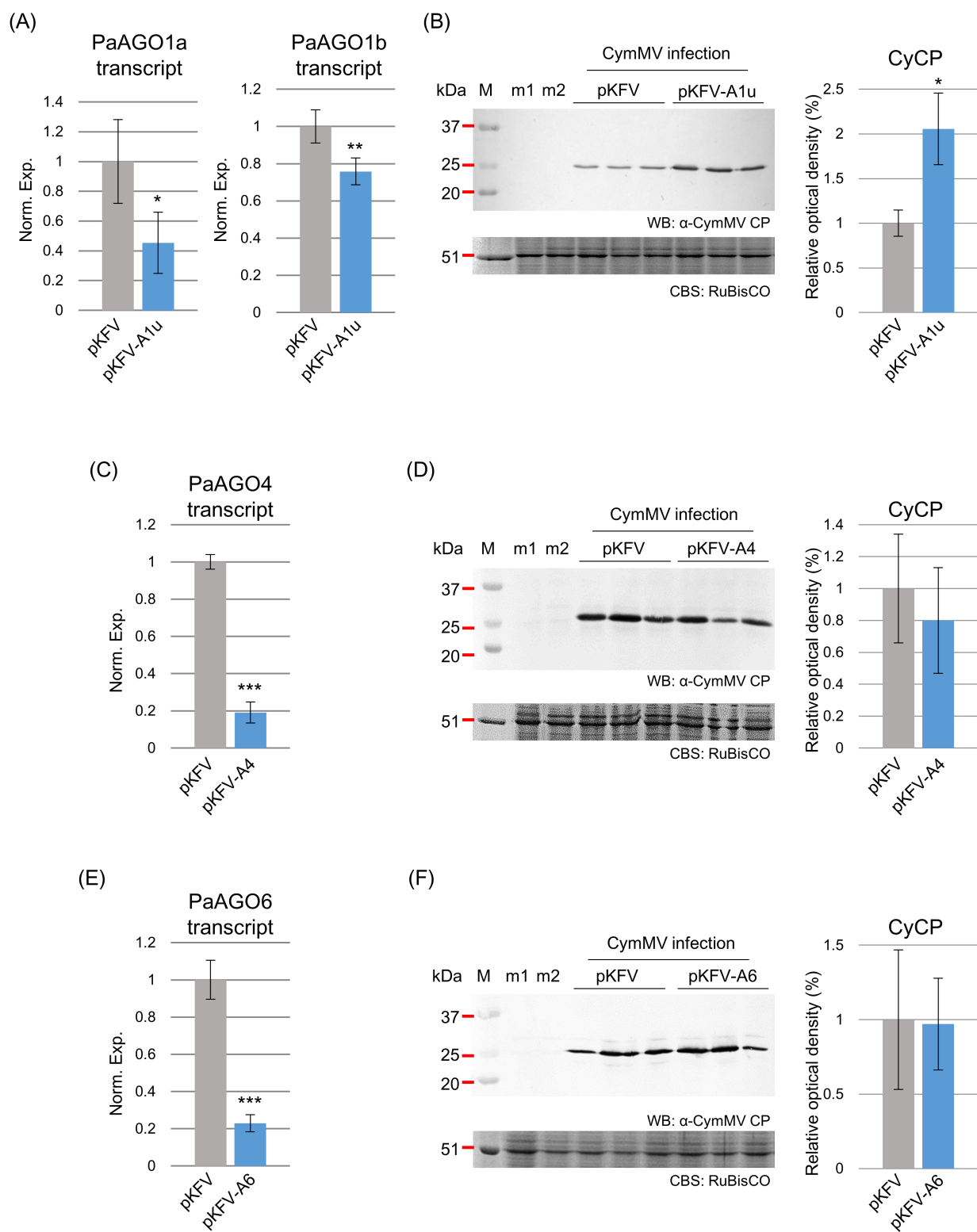

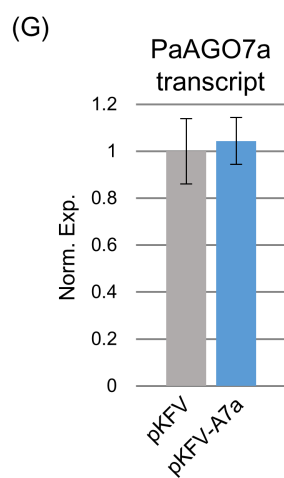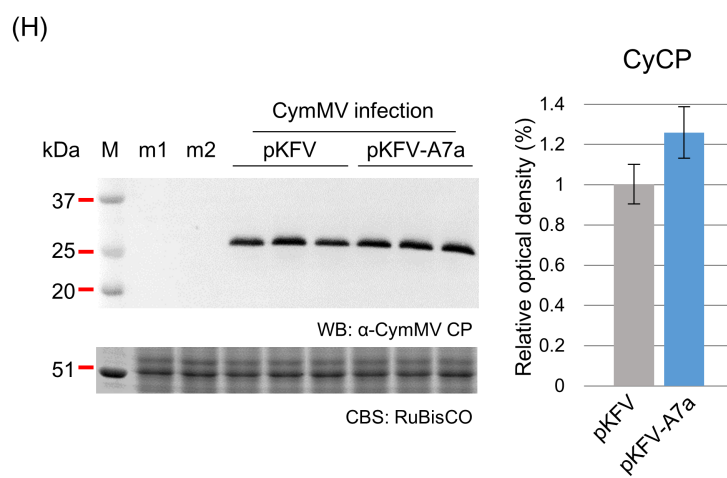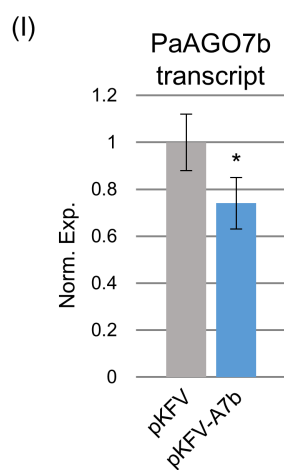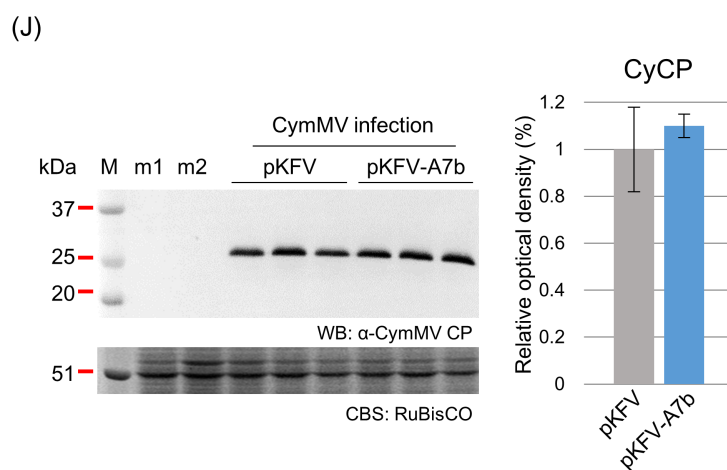

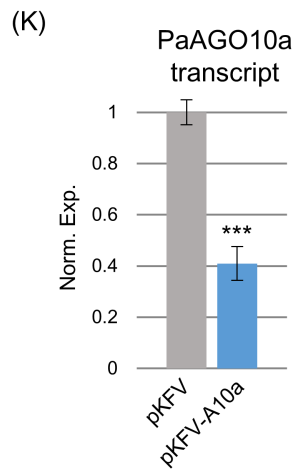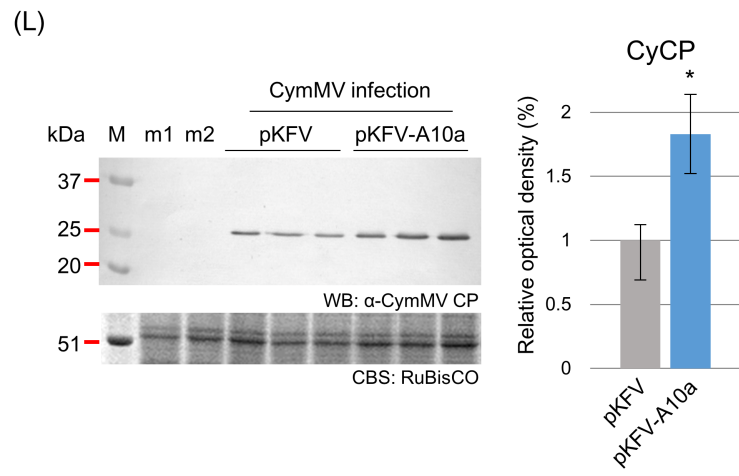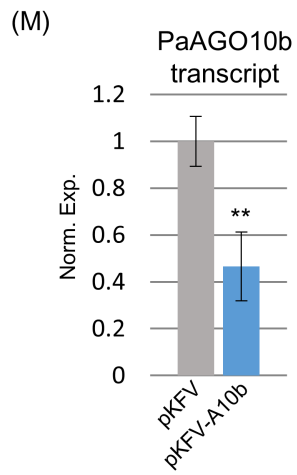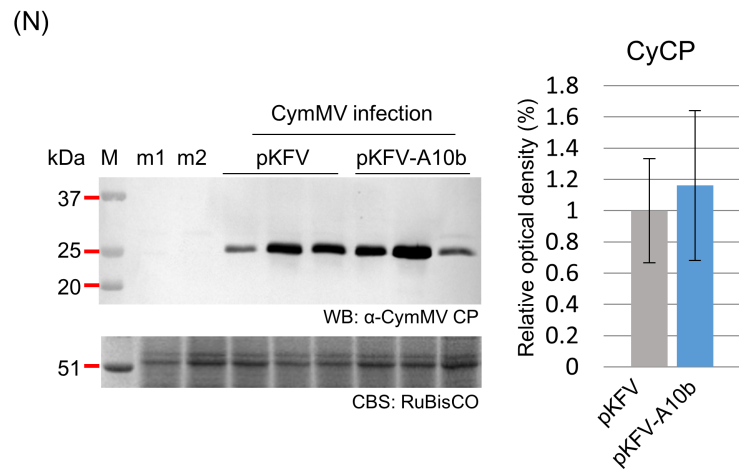

Supplement: Supplementary file 5 — FIGURE S5 Effects of knockdown of PaAGO1s/4/6/7s/10s on CymMV accumulation. Phalaenopsis aphrodite subsp. formosana leaves were infiltrated with Agrobacterium tumefaciens EHA105 harbouring the virus‐induced gene silencing (VIGS) constructs for silencing of respective PaAGOs (Table S1, as indicated on the top of each panel) or the empty vector pKFV 10 days before (−10 days postinoculation [dpi]) the inoculation of CymMV infectious constructs (0 dpi). Leaf samples were collected at 5 dpi. Total protein and RNA were extracted from inoculated leaves and the accumulation levels of CymMV and PaAGOs were analysed by western blot and quantitative reverse transcription PCR, respectively. The CymMV coat protein accumulation (CyCP) was quantified and plotted. The results of gene silencing of PaAGO1a and 1b (a,b), PaAGO4 (c,d), PaAGO6 (e,f), PaAGO7a (g,h), PaAGO7b (i,j), PaAGO10a (k,l), and PaAGO10b (m,n) are presented. Samples from leaves agroinfiltrated with pKFV plus pKn (m1) and respective PaAGO silencing construct plus pKn (m2) were used as negative controls [file MPP-22-627-s003.pdf]

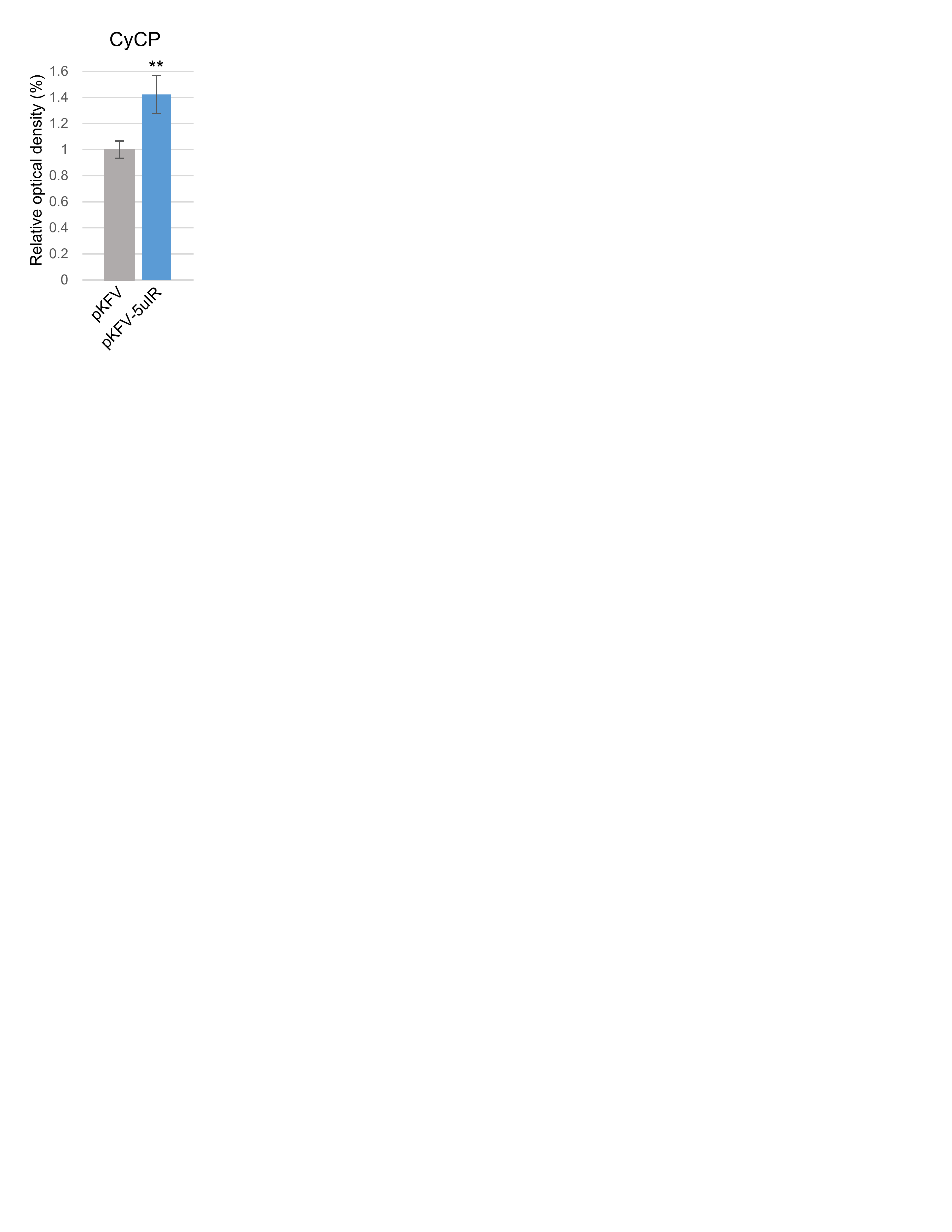

Supplement: Supplementary file 6 — FIGURE S6 Quantitative analysis of knockdown of PaAGO5s on CymMV accumulation. To determine specifically the effect of the silencing of individual PaAGO5s on CymMV accumulation, the data were extracted from the result presented in the western blot (left panel) of Figure 7d. The CymMV coat protein (CyCP) accumulation levels were determined by quantitative band density analysis. All bands were normalized to the accumulation level of RuBisCO [file MPP-22-627-s006.TIF]
